# Current Trends in Clinical Trials of Prodrugs

**DOI:** 10.3390/ph18020210

**Published:** 2025-02-04

**Authors:** Diogo Boreski, Valentine Fabienne Schmid, Priscila Longhin Bosquesi, Jean Leandro dos Santos, Cauê Benito Scarim, Viktor Reshetnikov, Chung Man Chin

**Affiliations:** 1 Laboratory for Drug Design (LAPDESF), School of Pharmaceutical Sciences, University of São Paulo State (UNESP), Araraquara 14800-903, Brazil; diogo.boreski@unesp.br (D.B.); priscila.bosquesi@unesp.br (P.L.B.); jean.santos@unesp.br (J.L.d.S.); caue.scarim@unesp.br (C.B.S.); 2 Departement Pharmazeutische Wissenschaften, Philosophisch-Naturwissenschaftliche Fakultät, Universität Basel, 4003 Basel, Switzerland; valentine.schmid@unibas.ch; 3 Advanced Research Center in Medicine (CEPAM), School of Medicine, Union of the Colleges of the Great Lakes (UNILAGO), Sao Jose do Rio Preto 15030-070, Brazil; 4 Department Chemistry and Pharmacy, Organic Chemistry II, Friedrich-Alexander-Universität Erlangen-Nürnberg, Henkestrasse 42, 91301 Erlangen, Germany; reshviktor1@gmail.com

**Keywords:** prodrugs, clinical trials, prodrug design, drug discovery

## Abstract

The development of new drugs is a lengthy and complex process regarding its conception and ideation, passing through in silico studies, synthesis, in vivo studies, clinical trials, approval, and commercialization, with an exceptionally low success rate. The lack of efficacy, safety, and suboptimal pharmacokinetic parameters are commonly identified as significant challenges in the discovery of new drugs. To help address these challenges, various approaches have been explored in medicinal chemistry, including the use of prodrug strategies. As a well-established approach, prodrug design remains the best option for improving physicochemical properties, reducing toxicity, and increasing selectivity, all while minimizing costs and saving on biological studies. This review article aims to analyze the current advances using the prodrug approach that has allowed the advance of drug candidates to clinical trials in the last 10 years. The approaches presented here aim to inspire further molecular optimization processes and highlight the potential of this strategy to facilitate the advancement of new compounds to clinical study phases.

## 1. Introduction

Prodrugs remain a cornerstone in medicinal chemistry, offering a versatile strategy for the development of novel therapeutic agents. This drug discovery strategy offers an effective solution for improving the physicochemical properties of drug candidates, particularly in addressing challenges encountered during the transition from preclinical to clinical trials. While modern drug discovery techniques like combinatorial chemistry and high-throughput screening generate novel chemical entities with potent pharmacological activity, many exhibit suboptimal physicochemical characteristics, necessitating chemical modification or formulation technologies for adequate performance. Thus, the prodrug approach facilitates the enhancement of diverse pharmacokinetic properties, including absorption, distribution, metabolism, and excretion (ADME), optimizing dissolution rates and lipophilicity without altering the drug target or indication or compromising pharmacological efficacy, while providing a streamlined approach to overcoming drug development hurdles [1,2,3].

**Figure 1:**
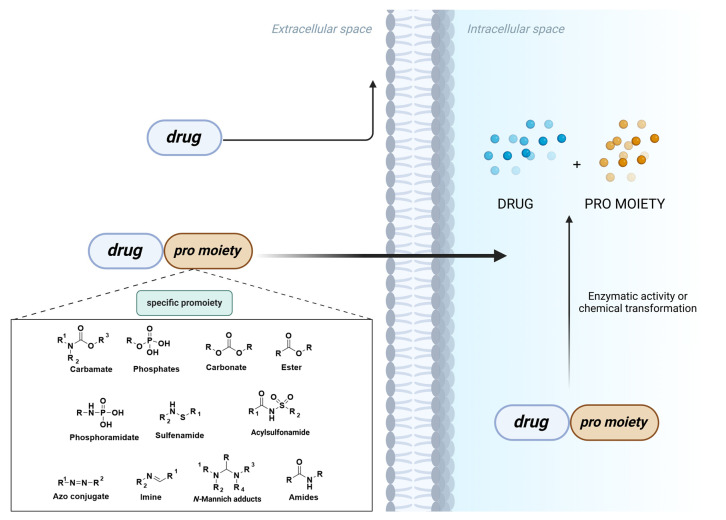
Scheme of the prodrug approach, highlighting key chemical modifications aimed at improving pharmacokinetic properties and overcoming physicochemical limitations.

In general, a prodrug can be defined as a biologically inert or inactive molecule, without pharmacological properties, which will be enzymatically or chemically activated within the human body. By incorporating pharmacologically inactive moieties—such as phosphates, esters, and amides—prodrugs optimize the chemical properties of active drugs (Figure 1), improving their bioavailability and reducing potential metabolic interference. The selection of the labile group in prodrugs carefully considers factors such as permeability, solubility, and hydrolysis kinetics to meet desired pharmacokinetic objectives, and selectivity to effectively target specific organs and/or tissues. This choice is crucial in identifying a successful prodrug candidate, since each chemical labile group exhibits a specific profile. For instance, functional groups like carbamates and amides can be effectively utilized to moderate rapid metabolism, owing to their slower hydrolysis kinetics [4,5,6].

**Figure 2:**
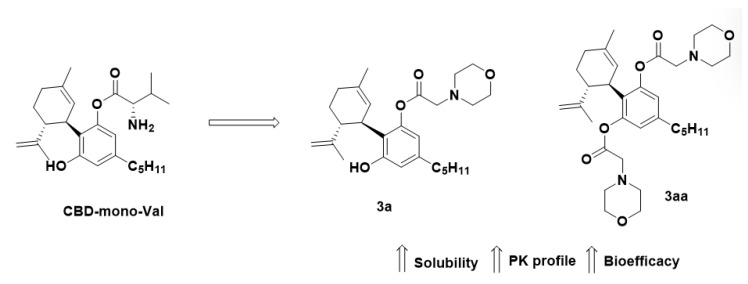
Examples of the chemical structure of CBD prodrugs in the preclinical stage.

Between 2008 and 2018, it was reported that the US Food and Drug Administration approved at least 30 prodrugs, representing over 12% of all approved small-molecule new chemical entities in that period [7]. Moreover, between 2014 and 2024, there was a remarkable increase in global patent filings for prodrugs, paralleling a rise in scientific publications mentioning the term “prodrug”. Over the past decade, an annual average of more than 4800 patent applications related to prodrugs were submitted, along with approximately 1261 scientific papers published each year that included this term. This represents a steady annual growth rate of around 1% in prodrug-related research. Particularly noteworthy was the increase of 468 new patent applications from 2016 to 2017. By 2023, the total number of prodrug patents reached a historic peak of 5730. However, in 2024, there was a decline of 1719 patent applications compared to the previous year. These trends, illustrated in Figure 2, emphasize the increasing relevance of prodrugs in pharmaceutical sciences and their potential to advance drug delivery and efficacy.

Considering the potential of the prodrug approach to optimize drug candidates, in this review article we analyze the clinical trial landscape for prodrugs over the past decade, highlighting those promising prodrugs that advance to clinical trials. Our aim will be concentrated on the discussion of the prodrugs strategy used for drug candidates in the clinical stage. For this analysis, we considered the period of 2014–2024, sourced from Clinical Trials (https://clinicaltrials.gov/, accessed on 12 June 2024), PubMed, ScienceDirect, Wiley Online Library, Scopus, and the Web of Science. Our investigation reveals that about 48 clinical trials were conducted (Table S1, Supplementary Materials), in which prodrugs were investigated for cancer, infectious diseases, central nervous system disorders, antifungal treatments, and anti-inflammatory therapies. The analysis of the use of the prodrug approach will be discussed based on successful examples, highlighting the main strategy pursued. The aim was to provide a general overview and present current advances by using this molecular optimization process.

**Figure 3:**
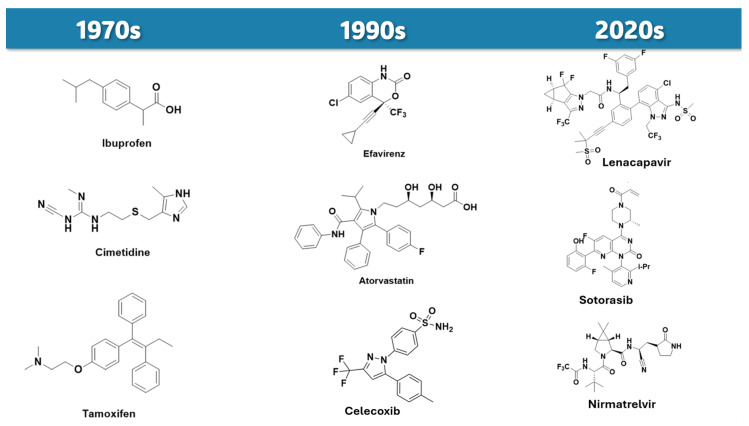
Relative complexity of drug structures over time.

A portion of this success may be attributed to scientific advancements and the development of new technologies that have facilitated selective tissue delivery, effectively addressing challenges related to solubility, permeability, and suboptimal pharmacokinetics. The conjugation of prodrugs with antibodies, nanobodies, aptamers, syderomycins, polymers, cell-penetrating peptides, lipids, polyethyleneglycol (PEG), and carbohydrates has greatly expanded the potential applications of this technology, fostering meaningful advancements in the use of this optimization approach [8,9,10,11,12]. In addition, over the past 40 years, there has been a significant increase in the complexity of drug chemical structures (Figure 3), resulting in compounds with low solubility and permeability. For these compounds, the prodrug approach is a powerful tool to improve physicochemical properties, allowing its advance in clinical trials.

In the literature there are innumerous examples of the prodrug approach being used to optimize promising compounds in both preclinical and clinical studies. One interesting example in the preclinical stage is cannabidiol (CBD). This phytocannabinoid is presented in different concentrations in cannabis plant species, and exhibits promising effects in the treatment of schizophrenia, epilepsy, Rett syndrome, anxiety, and sleep disorders, among others. As a hydrophobic molecule (logP +6.6), after oral administration CBD undergoes extensive first-pass metabolism, culminating in a low bioavailability ranging from 9 to 13% [13,14]. Singh Cham and colleagues [15] synthesized morpholinyl CBD-based prodrugs with better solubility (up to 24-fold) and pharmacokinetic profile (AUC ng.g/mL up to 4.3-fold) in comparison with the parental CBD (Figure 2). The water-soluble bisulfate salt and conjugation with the amino acid valine are also strategies used to improve the solubilization of CBD, are useful for treating ulcerative colitis and chronic inflammatory skin diseases, respectively [16]. Another interesting example of an FDA-approved drug is pegaptinib (Macugen^®^). This is a pegylated prodrug conjugated with the aptamer of the anti-vascular endothelial growth factor (VEGF) used in the treatment of age-related macular degeneration (AMD) [17], a common. disease that inflicts about 50% of all patients with visual impairment [18]. Pegaptinib is an RNA aptamer that binds to VEGF-165, responsible for ocular neovascularization and augmenting the permeability of ocular vessels. Aptamers are DNA or RNA oligonucleotides that bind with high specificity and affinity to proteins, and are commonly non-immunogenic and relatively small, thus offering advantages over antibodies. However, due to the instability of oligonucleotides (half-life less than 1 min), chemical modifications are mandatory, such as the substitution of 2-hydroxyl of the ribose moiety in pyrimidiones by an amino group or fluorine to ameliorate the nuclease resistance to hydrolysis and the replacement of 2′-hydroxyl position of purines by 2′-*O*-methyl [19]. The conjugation of 5′-linked 40-kDa PEG at t44-OMe improves the VEGF inhibition by up to 83% and also increases the half-life, prolongating the ocular contact. The PEG conjugation allows stability in human plasma for up to 18 h, and the residence time in the vitreous humor is up to 28 days after a dose of 0.5 mg intravitreous [20]. Clinical studies (VISION trial) showed that intravitreous administration of pegaptanib was able to reduce vision loss by up to 50% in the first year, stabilizing the vision in the second year [21].

## 2. Evolving Landscape of Prodrug Design

**Figure 4:**
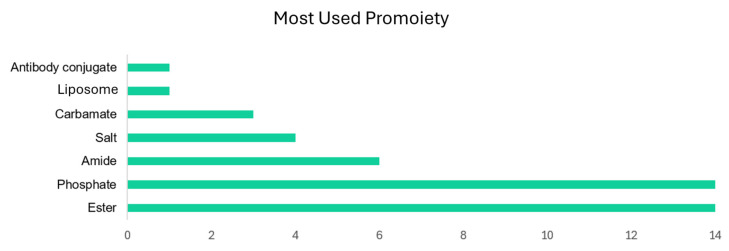
Most used promoieties in prodrug clinical studies in the past decade.

**Figure 5:**
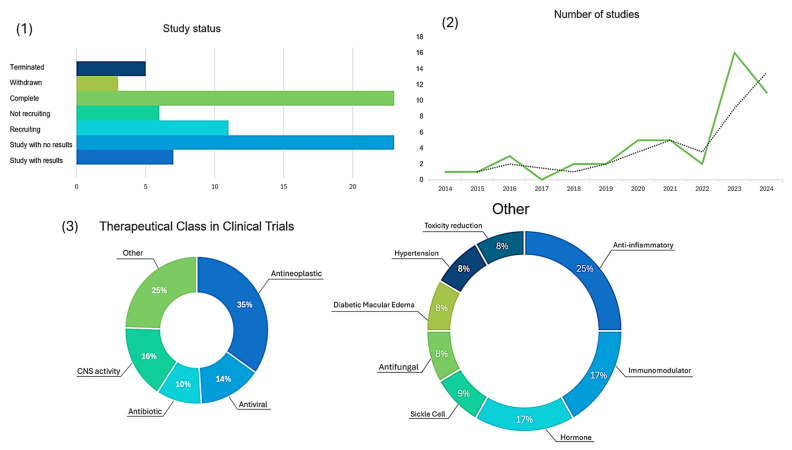
(1) Graph contains the status of all studies. (2) Graph shows the quantity of clinical studies per year over the past decade (green line) and its trend (black line). (3) Graph shows the percentage of clinical studies involving prodrugs by therapeutical age.

The challenge to discover novel chemical entities with satisfactory physicochemical, pharmacokinetic, and pharmacodynamic profiles in the drug discovery process can be modulated by the molecular optimization process. Significant advances in techniques for the identification and validation of molecular targets, enabled by modern technologies like genetic sequencing, metabolomics, proteomics, and more recently pharmacogenetics, along with in silico molecular modeling assisted by artificial intelligence and high-throughput screening, have allowed an appropriate rational for prodrug design due to the optimization of functional groups, carriers, and linkers employed in prodrug design. As a result of these advances, in the last decade diverse prodrugs have advanced to clinical trials (Figure 4 and Figure 5).

In this review, we summarize 48 clinical studies on new prodrugs tested over the past 10 years, which were identified by searching for the term “prodrug” and analyzing each study. Most of these studies are focused on discovering new treatments for cancer, representing 35% of all studies. This includes the treatment of lymphoma, refractory leukemia, advanced solid tumors, and lung cancer, Hsp90 inhibitor development, and some other cases. Following cancer treatments, drugs targeting the central nervous system (CNS) accounted for 16% of the studies, antiviral drugs accounted for 14%, and antibiotics accounted for 10%. Other therapeutic classes made up the remaining 25% of the studies, with anti-inflammatory drugs representing 25% of the tested prodrugs. The results are illustrated in Figure 5. Additionally, parameters such as study status and number of clinical studies were analyzed.

**Figure 6:**
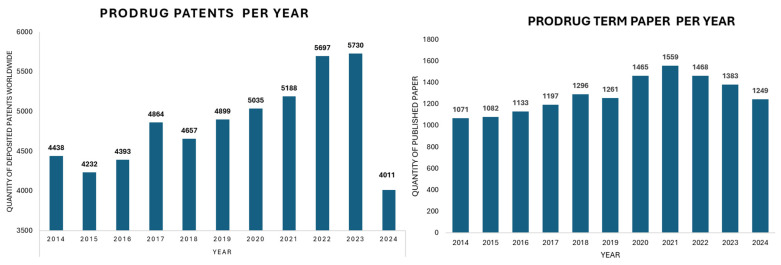
Quantity of scientific papers containing the term prodrug over the past decade (**right**). Quantity of deposited prodrug patents worldwide (**left**).

The number of clinical studies involving prodrugs has increased by an average of 8.9% per year since 2014. Figure 6 illustrates the growth in these studies since 2018, with 2 studies in 2018, 5 in 2020, 16 in 2023, and 11 in 2024. Among the evaluated compounds, 27% presented an ester moiety in their structure and another 27% featured a phosphate moiety. Amides, salts, and carbamates represent 12%, 8%, and 6%, respectively, of the prodrugs in clinical trials analyzed in this study. Other studies included liposomes, cleavable linkers, and antibody conjugates. These findings underscore the diversity and evolving nature of prodrug research in recent years, highlighting significant trends and structural preferences in the development of new therapeutic agents.

The selection of a promoiety is guided by the pharmacokinetic objectives of the designed molecule, particularly regarding ADME parameters. Each promoiety exhibits a unique metabolic profile. For instance, ester and phosphate groups are rapidly cleaved, resulting in a high rate of metabolism. Conversely, amides and carbamates display greater stability against enzymatic transformations, thus prolonging bond integrity. Salts, due to their ionic nature, can enhance aqueous solubility without requiring enzymatic conversion or bond cleavage, making them especially valuable in drug discovery and clinical applications. Unlike prodrugs, which often rely on enzymatic activation, salt formulations provide a straightforward approach to improve solubility and bioavailability. However, the nature of different ions in salt formation, whether organic acids, inorganic, metallic, or non-metallic, significantly impacts the pharmacokinetic profile of the prodrug, influencing half-life, solubility, and, consequently, absorption, distribution, metabolism, and excretion. Selecting the appropriate promoiety is thus critical for optimizing the pharmacokinetic properties of a prodrug. Although salts exhibit great importance in medicinal chemistry, they are not intrinsically considered prodrugs. However, they have been included in this review due to their significant potential to improve pharmacokinetic properties in the development of new drugs. Figure 4 illustrates the prevalence of the most used promoieties, highlighting the diverse strategies employed in prodrug design to enhance therapeutic efficacy and drug performance.

From 2014 to 2024, there has been a notable increase in global patent filings for prodrugs, which has paralleled the rise in scientific publications containing the term “prodrug”. On average, over the past decade, more than 4800 patent applications for prodrugs were submitted, alongside approximately 1261 scientific papers published annually that included the term. This reflects a steady growth rate of about 1% per year in prodrug-related research. Notably, from 2016 to 2017 there was an increase of 468 new patent applications. By 2023, the total number of prodrug patents reached a historic high of 5730. However, it is important to note that in 2024, there was a decrease of 1719 patent applications compared to 2023. These trends, underscore the growing significance of prodrugs in pharmaceutical sciences and their potential to enhance drug delivery and efficacy in pharmaceutical sciences.

In the next sections, examples of prodrugs that have advanced to clinical trials will be discussed, prioritizing some areas such as cancer, infectious diseases, central nervous system disorders, antifungal treatments, and anti-inflammatory therapies.

### 2.1. Antineoplastic Prodrugs

Cancer is a highly complex and multifaceted disease characterized by the uncontrolled and disordered growth of abnormal cells. It can affect almost any tissue or organ and is driven by a disruption in the normal regulatory molecular mechanisms that control and moderate cell division, growth, and death. Understanding cancer is complex because cancer involves delving into a large number of molecular mechanisms and multiple cellular pathways [22]. At its core, cancer originates primarily from genetic mutations or alterations that enable cells to evade normal growth restraints and acquire characteristics of malignancy. These alterations can affect various genes involved in cell cycle regulation, apoptosis, DNA repair, and signaling pathways that control cell proliferation and differentiation stimuli. Mutated genes such as oncogenes (e.g., epidermal growth factor receptor—EGFR), tumor suppressor genes (e.g., p53), and membrane receptors (e.g., fibroblast growth factor receptors) play pivotal roles in cancer development and severity [23,24,25]. Additionally, abnormalities in epigenetic modifications and alterations in the tumor microenvironment contribute to cancer progression and metastasis, also impacting treatment. Cancer is a significant global health challenge, with its incidence and prevalence varying across different regions and populations. According to the World Health Organization (WHO), cancer is one of the leading causes of morbidity and mortality worldwide, responsible for millions of deaths annually. The epidemiology of cancer is influenced by factors such as age, lifestyle (including smoking, dietary habits, and physical activity), environmental exposures (like radiation and carcinogens), infectious agents (such as hepatitis viruses and human papillomavirus), and genetic predisposition [26].

Treating cancer remains a hard challenge due to its heterogeneity, which means that tumors can differ widely even within the same type of cancer. This can be explained by the singularity of each patient and the high complexity and variety of molecular mechanisms occurring in each tumor. This heterogeneity affects treatment response and poses challenges for developing effective therapies and drug treatments. Traditional cancer treatments include surgery, radiation therapy, chemotherapy, and targeted therapy, each with its own set of benefits and a plethora of limitations. Emerging treatment modalities like immunotherapy, gene therapy, and personalized medicine more recently offer promising avenues but also present a big challenge in terms of cost, accessibility, and resistance development [22,26,27,28]. Table 1 shows antineoplastic prodrugs and their clinical trial phase status.

Resistance to antineoplastic medications can arise from various mechanisms within cancerous cells, such as modifying drug targets, overproducing drug efflux pumps, activating detoxification processes, reducing apoptosis susceptibility, enhancing DNA damage repair, and altering proliferation rates. Additionally, changes in the tumor microenvironment and immune responses can also foster resistance. Cancer cells often employ multiple strategies simultaneously, and differences between tumors underscore the need for personalized cancer therapies [25,29].

**Figure 7:**
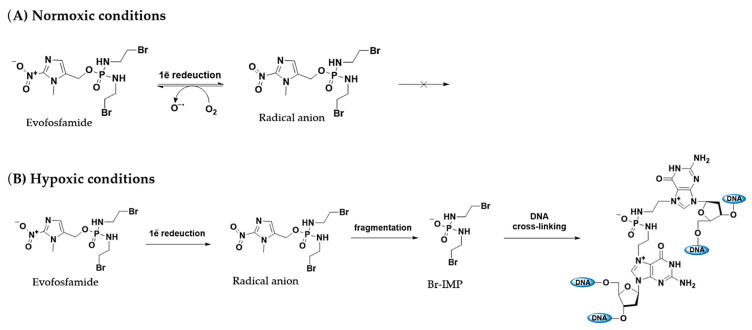
Evofosfamide mechanism of action. (**A**) Under normal oxygen conditions (normoxia), the radical anion form of the prodrug is rapidly oxidized by molecular oxygen, regenerating the original prodrug and producing superoxide. Due to this rapid oxidation, evofosfamide remains mostly inactive in oxygenated environments, as its activation is suppressed under these conditions. (**B**) Under severe hypoxic conditions (<0.5% O_2_), such as those found in poorly oxygenated tumor regions, the radical anion form of the prodrug undergoes reductive activation, leading to the release of the active cytotoxic agent, Br-IPM (bromo-isophosphoramide mustard), along with an azole byproduct. The released Br-IPM then alkylates DNA, forming both intrastrand and interstrand cross-links, which disrupt critical cellular processes and induce tumor cell death.

However, the primary goal of cancer treatment is to eliminate and halt the uncontrolled growth of a specific group of cells. This is typically achieved through a combination of drugs targeting various cellular processes and mechanisms. For instance, immunotherapy and drug therapy often involve using recombinant proteins to block membrane receptors responsible for growth stimuli. Some prodrugs, such as Evofosfamide, OBI3424, and CP506, become active through the complex sequence of reduction/degradation steps under cancer hypoxic conditions to further alkylate DNA via nucleophilic addition (Figure 7).

**Figure 8:**
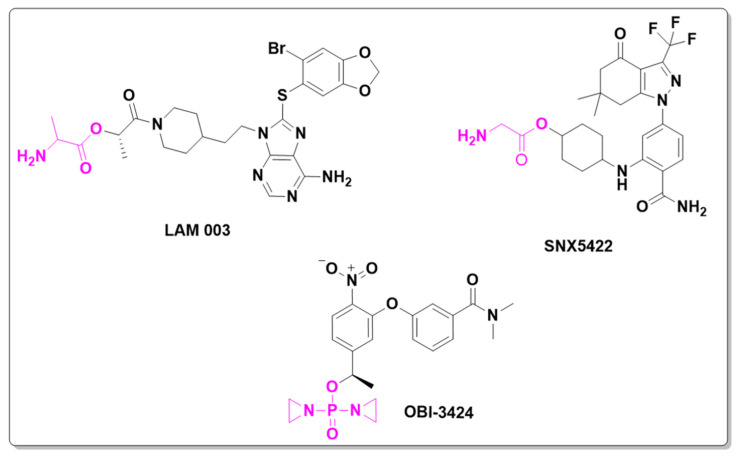
LAM003, SNX5422, and OBI-3424 prodrug chemical structures (magenta: moiety group).

Besides these, two other new small-molecule candidates have been identified and are currently in clinical trials focusing on cancer treatment. LAM003 and OBI-3424 (Figure 8) are prodrugs that entered Phase I clinical trials in 2020 and Phase II trials in 2024, respectively. LAM003 is a potent Hsp90 (heat shock protein 90) inhibitor that retains antileukemic activity against AML (acute myeloid leukemia) cells rendered resistant to FLT3 kinase inhibitors by mutation or stromal signaling [29].

This molecule is a purine analogue, linked to methylenedioxy and ethylpiperidine groups. It also contains an ester promoiety derived from L-valine, with a bioconversion index of ~90% in 24 h. Additionally, OBI-3424 is a highly selective prodrug converted by aldo-keto reductase family 1 member C3 (AKR1C3) into a potent DNA-alkylating agent. OBI-3424 has entered clinical testing for hepatocellular carcinoma and castrate-resistant prostate cancer and represents a potentially novel treatment for acute lymphoblastic leukemia (ALL). OBI-3424 is a nitrophenyl compound that includes a TEPA-moiety (triethylene phosphoramide) responsible for DNA alkylation [30,31].

Hsp90 plays a crucial role in tumor development and establishment. Inhibitors of the chaperone protein Hsp90 are of significant interest due to Hsp90’s central role in the maturation and maintenance of numerous proteins critical for tumor cell viability and growth [32]. SNX-5422 is another Hsp90 inhibitor whose clinical study was terminated in 2016. SNX-5422 is a methylamino ester prodrug and an oral formulation that demonstrates strong efficacy and tolerability, positioning it as a potential breakthrough therapy with broad applicability across a wide range of cancers [33].

These compounds belong to the class of haloalkylamines, a therapeutic class widely used in the treatment of certain types of cancer [34]. Alkylating agents are highly electrophilic chemicals that react with nucleophilic groups in DNA (specifically, the nitrogen atoms in DNA bases) through covalent bonding. These agents have been employed to disrupt DNA replication or transcription, ultimately leading to cell death. Their activity is primarily linked to their ability to cross-link DNA strands. However, due to their extreme reactivity, these agents often bind to other substrates (mainly proteins), making them less selective and quite toxic. In this way, the prodrug concept is helpful to reduce the unwanted activity, like cyclofosfamide, ifosfamide, mecloretamineand temozolomide, which are widely used in the clinic against different types of tumors [34].

These compounds feature two haloethyl groups (chlorine or bromine) attached to a nitrogen atom. These molecules are activated in the body, and this activation often involves enzymes in the liver or other tissues or by spontaneous activation, usually a pH-dependent process. Once activated, alkylating agents can form various types of DNA adducts, including monoadducts, where a single alkyl group is attached to a DNA base; cross-links, where two DNA strands are linked together by alkyl groups; intrastrand cross-links, where two bases on the same DNA strand are linked together; and interstrand cross-links, where bases on opposite strands of the DNA helix are linked together. These DNA adducts can interfere with DNA replication and transcription, leading to cell death. Within the molecular mechanism one of these haloethyl groups undergoes intramolecular cyclization, forming a highly reactive aziridinium ion. This aziridinium ion, due to its high electrophilicity, can react with nucleophilic groups in biological molecules, particularly with the N^7^ position of guanine, one of the DNA bases.

**Figure 9:**
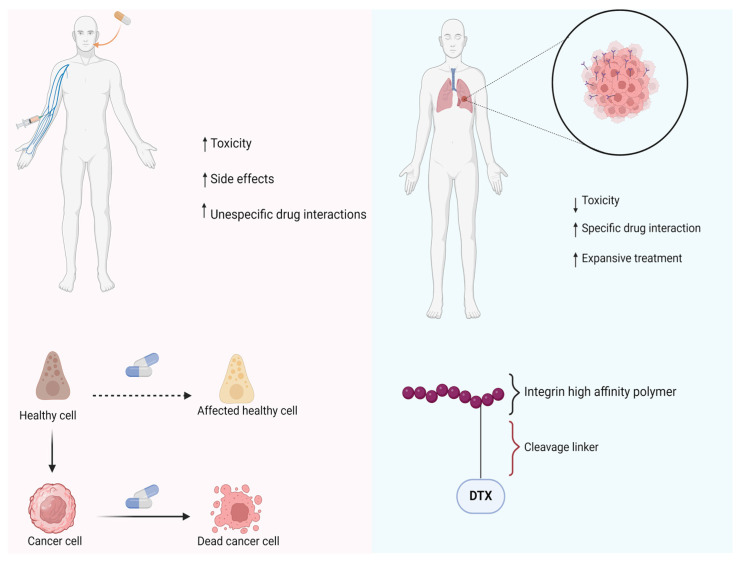
Comparison between conventional pharmacological therapies with less specific drugs (**left**) versus treatments with high-affinity ligands (**right**). Arrows indicate increase (↑) and decrease (↓).

Another type of prodrug that has gained prominence in recent years is the target-directed prodrug mediated by high-affinity ligands. In this type of prodrug, an active molecule is linked to a high-affinity ligand that specifically binds to receptors expressed in certain diseases. For example, RS-0139 is a delivery prodrug designed for the treatment of non-small cell lung cancer (NSCLC), which entered Phase I clinical trials in 2021 under the supervision of RS Research. This prodrug has a high affinity for integrin receptors, highly expressed on tumor cells, and is conjugated to docetaxel (DTX), also known as Taxotere^®^, an antineoplastic agent derived from Taxol. This prodrug design enables reduced toxicity and increased specificity in treatment (Figure 9).

Within the category of target-directed prodrugs, the high specificity is typically achieved by conjugation of popular drugs with the antibody, which serves as a vector to the target (for example, cancer cell). The challenges for the success of the system were the delivery of cytotoxic drugs from the conjugate into cancer cells. The first antibody conjugates were not prodrugs as they were not able to deliver the drug. As a way to solve this problem, a system named ADEPT (antibody–drug enzyme prodrug therapy) was proposed [35,36]. This method involves attaching an enzyme to an antibody that targets a tumor-associated antigen, allowing the enzyme to localize within the tumor. Once the enzyme has cleared from the bloodstream, a non-toxic prodrug is administered. The enzyme then activates the prodrug at the tumor site, transforming it into a powerful cytotoxic agent to kill cancer cells while minimizing damage to healthy tissues. This system was possible, but had serious problems of non-immunogenic enzymes to build the prodrug. To date, no ADEPT drug has reached the pharmaceutical market.

**Figure 10:**
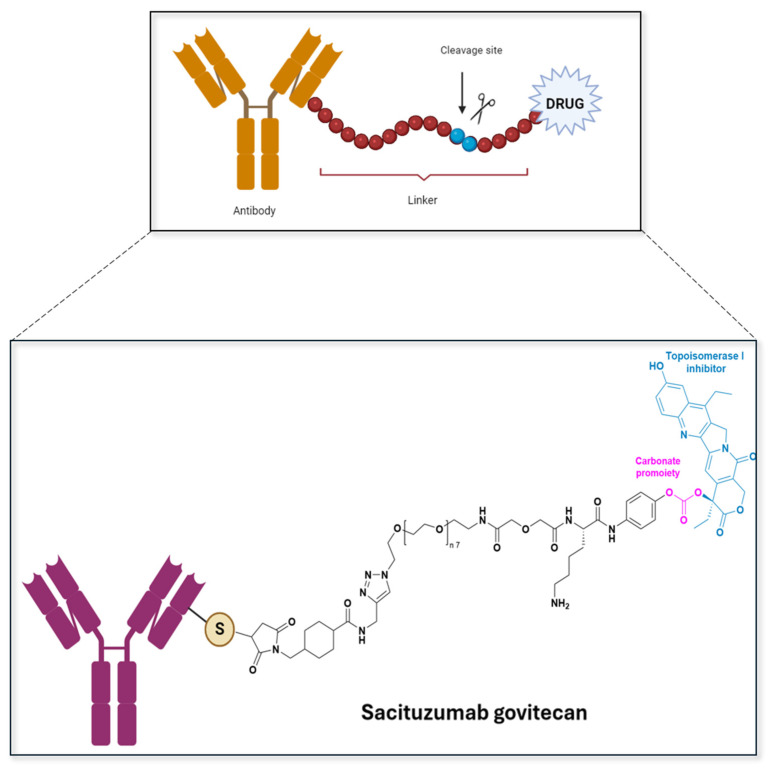
Antibody–drug conjugate scheme and Sacituzamab govitecan molecular structure (magenta: moiety group).

The goal of research with the antibody conjugate is to find a proper linker to construct an antibody–prodrug conjugate that is able to deliver the drug part to the decease site. In 2000, the first antibody–drug conjugate (ADC) Gemtuzumab ozogamicin—Mylotarg^®^—was approved by the FDA for the treatment of acute myeloid leukemia (AML). The ADC has a complex structure, encompassing recombinant antibodies that specifically bind to specific receptors, releasing the active drug at the tumor site. For example, IKS03 is a CD19-targeting antibody–drug conjugate with a novel linker-payload design for tumor-selective release. Using farnesyltransferase-catalyzed ConjuALL technology, IKS03 achieves site-specific conjugation to a DNA cross-linking agent, resulting in a homogeneous ADC with a drug-to-antibody ratio (DAR) of 2. To minimize systemic release of the potent payload in human plasma, IKS03 incorporates a linker cleavable by the lysosomal enzyme beta-glucuronidase. The payload is a prodrug with a protecting moiety also cleavable by the same enzyme. Thus, after CD19-dependent binding and uptake, IKS03 requires intracellular lysosomal processing in the target cell to release and activate the pyrrolobenzodiazepine (PBD) dimer payload, which induces DNA cross-linking, blocks DNA replication, and ultimately leads to tumor-selective cell death. IKS03 entered Phase I clinical trials in 2023. Figure 10 shows the antibody–drug conjugate scheme and Sacituzamab govitecan molecular structure, which was approved in USA in 2020 for treatment of metastatic triple-negative breast cancer.

Similarly, KGX101 is an Interleukin-12 (IL-12) prodrug that started Phase I clinical trials in 2023. IL-12 has been clinically limited due to its short half-life and dose-related toxicity, despite its potent activation of T cells and natural killer (NK) cells. To address these issues, KGX101 was designed as an IL-12 prodrug. It extends IL-12’s half-life by fusing its C-terminus with ADCC-impaired IgG1 Fc and concealing its N-terminus with receptor soluble domains IL-12Rβ1 and IL-12Rβ2 via tumor-specific protease-cleavable linkers. This design prevents systemic immune activation. In the tumor microenvironment, proteases cleave the linkers, enabling KGX101 to stimulate T cells and NK cells, reshape the tumor microenvironment, and show significant potential in cancer therapy. IFNγ (interferon γ) induction efficacy tests with PBMCs (Peripheral Blood Mononuclear Cells) from three donors confirmed KGX101’s masking effect, showing a reduction in activity of 300 to 2500-fold compared to unmasked IL-12-Fc [37].

In 2023, ADG206, an innovative prodrug version of a fully human monoclonal antibody (mAb) in the immunoglobulin G1 (IgG1) subclass, entered Phase I clinical trials. This prodrug specifically targets CD137, a co-stimulatory receptor, designed for advanced malignancy treatment. The Fc-enhanced IgG1 is covalently linked to a peptide mask, providing immunostimulant and antineoplastic properties. Upon administration, ADG206 is selectively activated within the tumor microenvironment, targeting and activating CD137 on various leukocyte subsets, including activated T lymphocytes and NK cells [38]. This activation enhances CD137-mediated signaling, induces cytokine production, and promotes T-cell-mediated anti-tumor immune responses. CD137, a surface glycoprotein in the tumor necrosis factor receptor superfamily, plays a critical role in T-cell proliferation, survival, and cytolytic activity [39].

T-1201 is an irinotecan (CAMPTOSAR^®^) prodrug that entered first-in-human clinical trials Phase I in 2021, under the responsibility of Taivex Therapeutics, Inc. This prodrug employs a target delivery system as a small molecule drug conjugate (SMDC), demonstrating potential for multiple anti-cancer indications. T-1201 operates through a mechanism like antibody–drug conjugates. It specifically targets the cell surface marker phosphatidylserine to accurately identify cancer cells. Its efficacy has been demonstrated in vivo across various xenograft animal models, including colorectal, pancreatic, prostate, lung, breast, and liver cancers. In animal studies, T-1201 has shown significantly higher potency and lower toxicity compared to the marketed drug irinotecan. Interestingly, irinotecan is already a carbamate prodrug.

Table 2 shows the success of ADC prodrugs, which can be observed by the robust number of ongoing clinical trials. In the last decade, 804 studies involving ADC were carried out with 329 studies finalized. This type of prodrug is not found in the database “clinical trials” search using the term “prodrug”, but antibody–drug conjugate or ADC.

**Figure 11:**
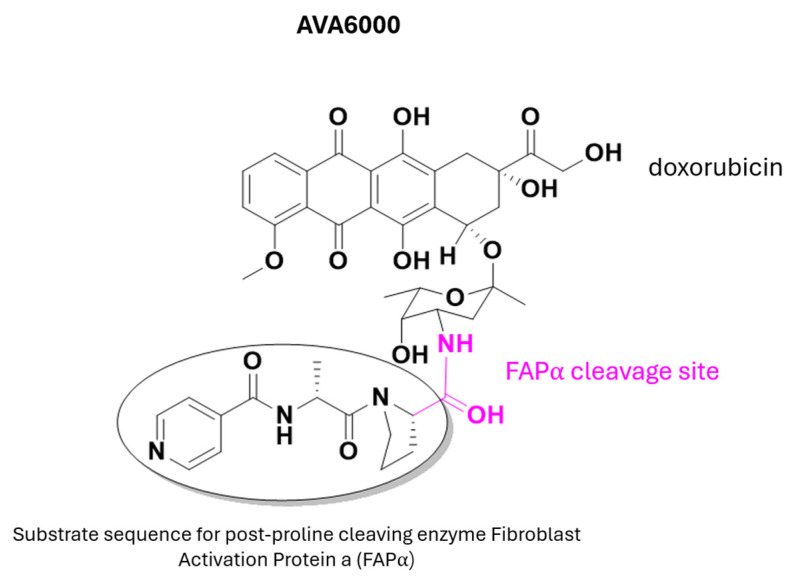
AVA6000 prodrug chemical structure. (magenta: moiety group).

Among break-bond prodrugs, AVA6000 (Figure 11) is a promising antineoplastic consisting of two moieties: a doxorubicin molecule linked to an FAPα (fibroblast-activated protein α) binding moiety. This prodrug, which began Phase I clinical trials in 2021, aims to treat patients with locally advanced and/or metastatic solid tumors. The overexpression of FAPα on the surface of stromal cells in human tumors (compared to normal tissue) provides the premise for developing prodrugs that remain inert until activated by the enzymatic activity of FAPα within the tumor microenvironment [40].

**Figure 12:**
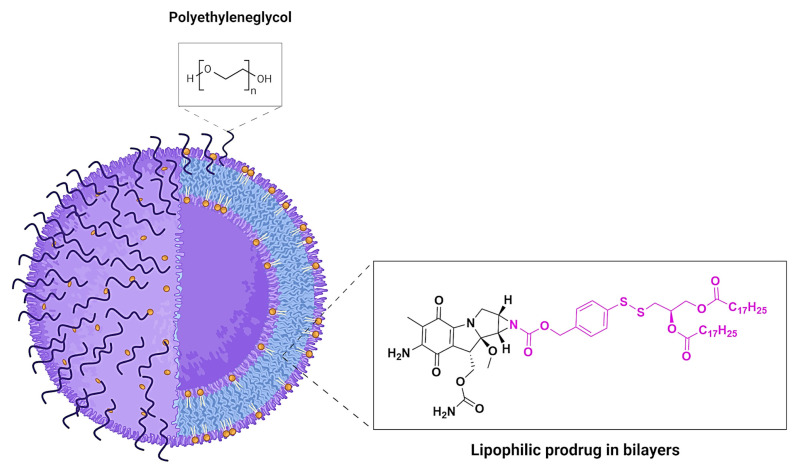
PROMITIL-mitomycin liposome containing on its surface a PEG layer and mitomycin C prodrug chemical structure (magenta: moiety group).

Recently, a new prodrug concept has been developed, PROMITIL-mitomycin, a lipid-based liposomal prodrug containing mitomycin C (Figure 12). This compound, isolated from *Streptomyces caespitosus* or *Streptomyces lavendulae*, is part of a family of aziridine-containing natural products with strong anti-tumor and antibiotic activity. Its mode of action involves DNA cross-linking, which disrupts protein synthesis and cell replication. However, the clinical use of mitomycin C has been limited due to its toxicity profile, particularly causing prolonged bone marrow suppression and off-target tissue damage. Liposomes are widely used in targeted drug delivery systems. They can encapsulate both hydrophilic and lipophilic agents, enhancing drug stability, bioavailability, and targeted release. Numerous liposomal formulations have been approved for cancer therapy, in which drugs are generally encapsulated in the aqueous core or stabilized by transmembrane ion gradients. An advanced strategy in liposomal delivery involves chemically modifying drugs into lipidic prodrugs that integrate into the liposomal bilayer, further enhancing stability and drug retention. For example, PEGylated liposomal doxorubicin (PLD; Doxil™ and its generics), is one of the most successful nanomedicines in oncology, demonstrating increased efficacy and reduced systemic toxicity. The development of lipid–drug conjugates and lipophilic prodrugs has significantly advanced nanomedicine, with multiple formulations approved for clinical use in cancer therapy [41].

### 2.2. Antibiotic Prodrugs

Antibiotics represent a revolutionary technology for human health, as the ability to specifically disrupt the life cycle of a pathogen is a key factor in developing safe treatments. The discovery of penicillin in 1928 transformed the approach to bacterial infections, as the administration of this drug, which is still in use today, can effectively eliminate some infectious conditions [42]. Antibiotics represents a generic term for a wide variety of compounds that act through different mechanisms of action, and are separated into classes, such as aminoglycosides, β -lactams, quinolones, glycopeptides, oxazolidinones, sulfonamides, tetracyclines, and lipopeptides. However, β-lactamases are a group of enzymes in bacterial cells that hydrolyze the amide bond of the four-membered β-lactam ring, present in drugs like penicillin, thus marking the primary resistance mechanism [43]. Nonetheless, due to the crisis of antibiotic resistance, the discovery of new compounds to withstand bacterial infections is essential [44]. Challenges such as antibiotic pharmacokinetics represent a significant issue, given that the primary available class of antibiotics, β-lactams and carbapenems, is sensitive to gastric pH. This necessitates strategies based on molecular modifications or pharmaceutical technology adjustments, such as acid-resistant capsules to achieve orally bioavailable drugs [45]. Recently, Reddy et al. reported new boron-derivate β-lactamase inhibitors, such as QPX7831. QPX7831 demonstrated 100% oral bioavailability in non-human primates and water solubility of 10.6 mg/mL. This prodrug, an ester from isobutyric acid, belongs to the same class of the non-β-lactam β-lactamase inhibitors such as Vaborbactam (Vabomere™). The compound is currently in Phase I clinical trials [46].

Similarly, SPR994, a prodrug known as Terbipenem, is a carbapenem hydrobromide salt esterified with a pivoxil promoiety. Developed by GlaxoSmithKline for oral administration, it is currently in Phase III clinical trials. This new prodrug is indicated for urinary tract complications resistant to fluoroquinolones. Clinical cure was observed at the test-of-cure visit in 93.1% of the patients treated with tebipenem hydrobromide [47].

The addition of ester groups in the design of prodrugs helps to circumvent undesirable aspects such as acid ionization, which impedes cellular membrane passage, and introduces moieties that enhance solubility. Esters are easily metabolized by plasma and intestinal esterases [48]. Another group easily cleaved by plasma enzymes is that of phosphate esters, which have been widely explored in prodrug development, with important representatives like fospropofol, fosfluconazole, fosphenytoin, and fosamprenavir. Talley et al. conducted a study on a first-in-class aminobenzimidazole bacterial DNA gyrase (GyrB) inhibitor—SPR720 or Fobrepodacin. This prodrug is a phosphate prodrug of SPR719, designed to treat nontuberculous mycobacterial pulmonary disease (NTM-PD) and pulmonary tuberculosis. The Phase I study evaluated six different dosage administrations ranging from 100 mg to 2 g over the maximum duration of 14 days. The authors concluded that the phosphate prodrug is completely hydrolyzed to its active form by alkaline phosphatase enzymes [49]. Another phosphate prodrug, Afabicin or DEBIO 1450, an acrylamide pyridine-δ-lactama benzofuran, is currently being tested for *Staphylococcus* spp. infections. The drug’s mechanism of action involves the inhibition of fatty acid synthesis by inhibiting the FabI enzyme. DEBIO 1450 offers a unique mechanism of action with a focused spectrum targeting staphylococci, making it a promising investigational therapy for challenging staphylococcal infections [50,51]. Afabicin is currently in Phase II clinical trials.

**Figure 13:**
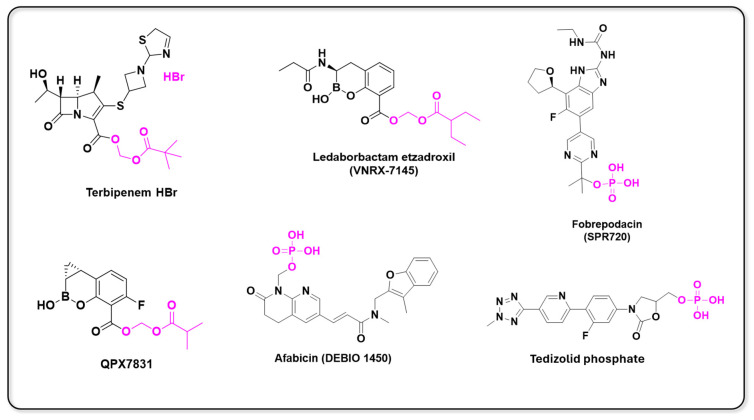
Terbipenem HBr, ledaborbactam etzadroxil, fobrepodacin, afabicin, QPX7831, and tedizolid phosphate prodrug chemical structures (magenta: moiety group).

The development of Ledaborbactam etzadroxil, an active and suitable for oral administration of benzoxaborinine-based β-lactamase inhibitors, was possible due the ester prodrugs synthesis. The protection of carboxylic acid by the ester promoiety is a powerful tool that is widely used in medicinal chemistry; an example is Enalapril, an antihypertensive drug, and angiotensin-converting enzyme inhibitor, composed of the amino acids L-proline and L-alanine. After enzymatic conversion, Enalapril releases its active form, Enalaprilat. Ledaborbactam etzadroxil is a pivoxyl ester prodrug of Ledaborbactam. The authors synthesized different ester prodrugs using various alkyl and acyloxy ester moieties; however, the pivoxyl and 3-pentyl acyloxy prodrugs demonstrated the highest oral bioavailability and were tested. Currently, the pivoxyl compound is in Phase I clinical trials [52]. On the other hand, QPX-7831 is a butyric acid ester derivative prodrug from QPX7728. QPX7728 is a new β-lactamase inhibitor with a broad spectrum of inhibitory activity that includes not only essentially all class A and class C enzymes from Enterobacterales, but also class B metalloenzymes such as NDM-1 and class D enzymes from *Acinetobacter* spp. While the oral bioavailability of this agent is 50% in rats and dogs, it is only 20% in monkeys. Rather than face the potential risk of low or variable oral bioavailability of QPX7728 in humans, we sought to identify a prodrug of QPX7728 showing consistently high oral bioavailability in multiple species [46]. Tedizolid or Sivextro^®^ is an FDA-approved phosphate prodrug indicated for resistant infection treatment. This compound started Phase I clinical trials in 2016 to evaluate its oral and intravenous safety in patients from age 2 to under 12. Tedizolid, a small molecule containing four ring moieties, is an oxazolidinone drug composed of another three rings: the methyltetrazole ring, pyridine ring, and a fluorobenzyl ring. This small molecule works by inhibiting protein synthesis by bacterial ribosomes [53]. Ledaborbactam and QPX7728 incorporate ethyl 2-ethyl butyric acid and isobutyric acid as promoieties, respectively. Preclinical studies using these organic acids have shown that they are the most effective and safest options, with bioavailability ranging from good to excellent. The use of short-chain carboxylic acids enhances water solubility, ensures good bioavailability, and allows for easy cleavage without releasing acids with significant toxic potential. Figure 13 shows the prodrug structure of some antibiotic prodrugs.

### 2.3. Antiviral Prodrugs

Since their discovery in 1892, viruses have been a contentious subject within the scientific community. The debate over whether viruses should be classified as living organisms continues to divide scientists today. Despite this uncertainty, viruses have consistently posed serious public health threats, with viral infections claiming millions of lives prematurely [54,55,56]. Among these, the discovery of the human immunodeficiency virus (HIV) in 1983 introduced a significant scientific and societal challenge.

In 1987, Zidovudine (AZT) became the first drug approved by the Food and Drug Administration (FDA) for HIV therapy, marking the beginning of a new era in antiretroviral treatment. As a reverse transcriptase inhibitor, Zidovudine paved the way for Antiretroviral Therapy (ART), an effective approach that transformed the previously lethal acquired immunodeficiency syndrome (AIDS) into a chronic, manageable condition. Today, HIV treatment is characterized by modern drugs such as tenofovir, dolutegravir, and emtricitabine, which offer high efficacy, safety, and low toxicity. Nonetheless, the search for new antiretroviral agents remains critical. In 2012, Nowicka-Sans and colleagues [57] introduced BMS-663068, also known as Fostemsavir—a phosphonooxymethyl prodrug of Temsavir. This innovative small-molecule attachment inhibitor targets the HIV-1 gp120 protein, blocking its binding to CD4(+) T cells. By 2018, Fostemsavir had progressed to Phase I clinical trials to assess its pharmacokinetics and safety, and is now recommended for HIV-1 treatment [58].

Similarly, MK-8510, a prodrug of MK-8558, entered Phase I clinical trials in 2023 in a study conducted by Merck, though molecular data for MK-8558 remain unavailable. A Phase IV clinical study led by Jidong Jia, Inc., is currently evaluating the use of Tenofovir alafenamide (TAF) for liver fibrosis. TAF, a purine analogue drug, is already FDA-approved as a prodrug of tenofovir for HIV treatment. Likewise, bemnifosbuvir hemissulfate (AT527) is an orally bioavailable direct-acting antiviral and purine nucleotide prodrug with potential efficacy against various RNA viruses. In its active form, this prodrug inhibits the viral RNA-dependent RNA polymerase, resulting in the termination of viral RNA transcription, reduced viral RNA production, and inhibition of viral RNA replication. This compound is in Phase II clinical trials for Hepatitis C Virus (HCV) infection, targeting the nonstructural protein 5B (NS5B) polymerase, in a study initiated by Atea Pharmaceuticals, Inc. in 2020.

Fosclevudine alafenamide (ATI2173), a phenoxyphosphonamide prodrug, is currently under investigation for its effects on Hepatitis B Virus (HBV) patients, with Phase I clinical trials having commenced in 2021 by Antios Therapeutics, Inc. This clevudine prodrug functions by inhibiting viral plus-strand DNA synthesis, similar to clevudine. The study aims to assess the safety, tolerability, pharmacokinetics, and antiviral activity of this phosphate prodrug.

The SARS-CoV-2 virus triggered a global health crisis in 2020 and continues to circulate. Although the COVID-19 pandemic was controlled through mass vaccination, a major achievement in vaccine technology and clinical research, there remains no consistent drug treatment. Paxlovid^®^ (nirmatrelvir) is currently the only FDA-approved treatment for severe SARS-CoV-2 infection. Additionally, PF-07304814, a phosphate prodrug of PF-00835231 developed by Pfizer Inc., entered Phase I clinical trials in 2021. This study, which is the second clinical administration of PF-07304814, aims to evaluate the safety, tolerability, and pharmacokinetics of escalating single doses of the prodrug in healthy adult participants [59].

**Figure 14:**
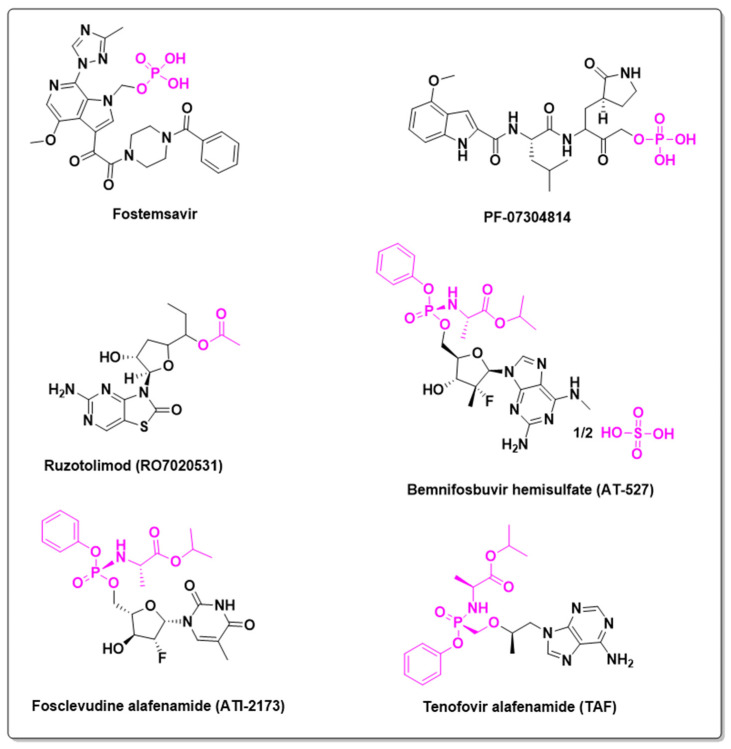
Fostemsavir, PF-0035231, ruzotolimod, bemnisfosbuvir hemisulfate, fosclevudine alafenamide, and tenofovir alafenamide prodrug chemical structures (magenta: moiety group).

Ruzotolimod (RO7020531), a thiazolopyrimidinone derivative with TLR7 agonist properties, is potentially active against both HBV and SARS-CoV-2 infections. Currently, it is being evaluated in patients with chronic HBV infection in a study funded by Hoffmann-La Roche Inc. Preliminary findings indicate a favorable pharmacokinetic profile, with doses of at least 100 mg being safe and well tolerated. On-target pharmacodynamic effects were observed, with response levels plateauing between doses of 140 mg and 170 mg. However, further studies are required [60]. Chemical structures of some antiviral prodrugs are shown in Figure 14.

### 2.4. Central Nervous System Active Prodrugs

The central nervous system (CNS) is an extraordinarily complex and uniquely structured organ, posing substantial challenges for developing new treatments. It is primarily regulated by small neuroactive molecules called neurotransmitters, which control almost all neurological processes, including emotions, motor control, self-regulation, learning, and various physiological functions. The global market for CNS disorder therapies was valued at around USD 50 billion in 2001, increased to USD 169 billion in 2021, and is projected to grow further, reaching USD 210 billion by 2026 [61,62].

Due to its complex nature, the CNS is naturally protected by the blood–brain barrier (BBB), a highly selective and tightly regulated filter that controls and restricts the movement of substances between the bloodstream and the brain parenchyma [63]. Consequently, the BBB is a significant obstacle in drug discovery and design for brain-targeted therapies.

BBB penetration occurs through two primary mechanisms: passive transport and active transport. Passive transport, often considered the most important route, is primarily influenced by a drug’s lipophilicity. However, it is now well established that increased lipophilicity often comes with challenges such as poor solubility, reduced metabolic stability, and a heightened risk of nonspecific, off-target effects, which may lead to undesirable toxicological outcomes [64]. Conversely, active transport is mediated by membrane receptors that recognize specific molecules, such as dopamine, serotonin, and glutamate [65]. This feature has been exploited in drug discovery to develop CNS-targeting drugs, including antiparkinsonian and anti-Alzheimer’s agents like Levodopa and Carbidopa, which are recognized and transported across the BBB by these receptors. Consequently, CNS-active drugs remain a key focus within the pharmaceutical industry. For instance, BMS-986465, an orally available TYK2 (tyrosine kinase 2) inhibitor, can penetrate the CNS and is currently in Phase I clinical trials for neuroinflammation and multiple sclerosis. This study, which began in February 2024 under the direction of Bristol Myers Squibb, involves the active metabolite of BMS-986465, an *N*-demethylated compound on the triazole ring.

Another example is Troriluzole, a prodrug of Riluzole (Rilutek^®^), investigated for Alzheimer’s disease [66], glioblastoma [67], obsessive-compulsive disorder [68], and spinocerebellar ataxia type III [69]. Its mechanism involves inhibiting voltage-dependent sodium channels, reducing synaptic glutamate by increasing its uptake and inhibiting its release. Troriluzole, a trifluoromethoxy benzothiazole derivative, has a cleavable polyamide segment that prolongs its half-life and enhances water solubility.

Psilocybin and psilocin are naturally occurring compounds found primarily in fungi. Due to their structural similarity to serotonin, they have strong effects on the brain. Psilocybin, a natural phosphate derivative found in Psilocybe cubensis (commonly known as magic mushrooms), is rapidly metabolized by specific phosphatases into its active form, psilocin. These compounds are hallucinogenic, as they act as agonists for 5-HT2A serotonin receptors [70]. Their use dates to Aztec civilizations, where they were consumed in sacred rituals in Mexico and Guatemala [71]. Despite being prohibited in many countries, recent studies have shown the potential therapeutic benefits of these substances. In 2023, Lobe Sciences Ltd. conducted a Phase I clinical trial to evaluate the bioavailability, safety, tolerability, and pharmacokinetics of 2 mg of psilocybin and psilocin mucate salt. This “first-in-man” study aimed to assess the compounds’ effects on mental state and anti-anxiety properties.

Antidepressants are medications used to treat depressive disorders by balancing neurotransmitters such as serotonin, norepinephrine, and dopamine in the brain. The first approved antidepressant was iproniazid (Marsilid^®^), a monoamine oxidase inhibitor (MAOI), in the 1950s. Since then, various classes of antidepressants have been developed, including tricyclic antidepressants (TCAs) (e.g., amitriptyline), selective serotonin reuptake inhibitors (SSRIs) (e.g., fluoxetine, sertraline), and serotonin-norepinephrine reuptake inhibitors (SNRIs) (e.g., venlafaxine, duloxetine). SSRIs are the most prescribed due to their efficacy and fewer side effects compared to older classes [72]. Recently, a new candidate, LY03005 (also known as Ansofaxine or Ruoxinlin^®^), began Phase I clinical trials in 2014. This prodrug, a toluyl ester of venlafaxine, advanced to Phase II in 2022 and Phase III in 2023 under Luye Pharma. Studies led by Weifeng Mi and colleagues in 2022 and 2023 assessed the safety and efficacy of this prodrug [73,74]. In November 2022, toludesvenlafaxine was approved for depression treatment in China.

In the treatment of depressive disorders, ABX-002, a methylamide prodrug, began Phase I clinical trials in 2023 and progressed to Phase II in 2024, conducted by Autobahn Therapeutics Inc. ABX-002 is an orally administered, potent, and selective thyroid hormone beta (TRβ) agonist with enhanced brain targeting. It demonstrates target engagement in brain regions associated with depression and shows reduced peripheral effects compared to synthetic thyroid hormone T3 at its therapeutic dose. ABX-002 is intended as an adjunctive treatment for major depressive disorder, potentially enhancing and amplifying the effects of antidepressants by boosting monoaminergic signaling in the brain.

Fencamfamine, an aminoalkane drug originally introduced by Merck in the 1960s, acts as an indirect dopamine D2 receptor agonist. It stimulates dopamine release like amphetamines, albeit with significantly lower potency—approximately 10 times less potent than dexamphetamine [75,76]. This medication is primarily used to address daytime fatigue, difficulty concentrating, and lethargy associated with depressive conditions. It is particularly suitable for patients with chronic illnesses due to its favorable safety profile. However, fencamfamine is contraindicated in individuals with heart conditions such as angina pectoris or decompensated heart failure, glaucoma, hyperactivity, hyperthyroidism, or those undergoing treatment with monoamine oxidase inhibitors

PRX-P4-003 is a highly lipophilic carbamate prodrug from (-)-fencamfamine that started Phase I clinical trials in 2021 to evaluate the microdose pharmacokinetics in healthy volunteers. The study was conducted by Praxis Bioresearch, LLC using 40 μg of (-)-fencamfamine and 100 μg of fencamfamine prodrug designed to provide a once-a-day drug to treat apathy caused by Alzheimer’s disease. Specifically, the objective of the molecular design of PRX-P4-003 is to deliver an active isomer of fencamfamine selectively bioavailable via the oral route, thus differentiating it from currently FDA-approved stimulants.

Opioids are a class of drugs primarily used for pain relief, working by binding to µ opioid receptors in the brain and spinal cord to reduce pain perception. This class includes natural compounds like morphine, derived from the opium poppy and recognized as one of the most potent analgesics available [77]. Morphine has been widely used to manage both acute and chronic pain, particularly in cases of cancer and post-surgical pain. Hydrocodone, a semi-synthetic opioid derived from codeine, is commonly prescribed for moderate to severe pain and as a cough suppressant. Despite its effectiveness, hydrocodone presents significant clinical challenges due to its high potential for addiction and abuse, contributing notably to the opioid crisis. Long-term use can lead to tolerance, physical dependence, and opioid use disorder. Furthermore, overdose poses a serious risk of respiratory depression, and constipation is a frequent side effect of prolonged opioid use [78].

In 2022, Elysium Therapeutics, Inc. initiated a Phase I clinical trial to evaluate the safety, tolerability, and pharmacokinetics of overdose-protected hydrocodone prodrugs compared to hydrocodone bitartrate hemipentahydrate (HCBT) after single oral doses in healthy adult subjects, under both fasted and fed conditions with naltrexone blockade. This study used hydrocodone acetate salt as the investigational prodrug.

**Figure 15:**
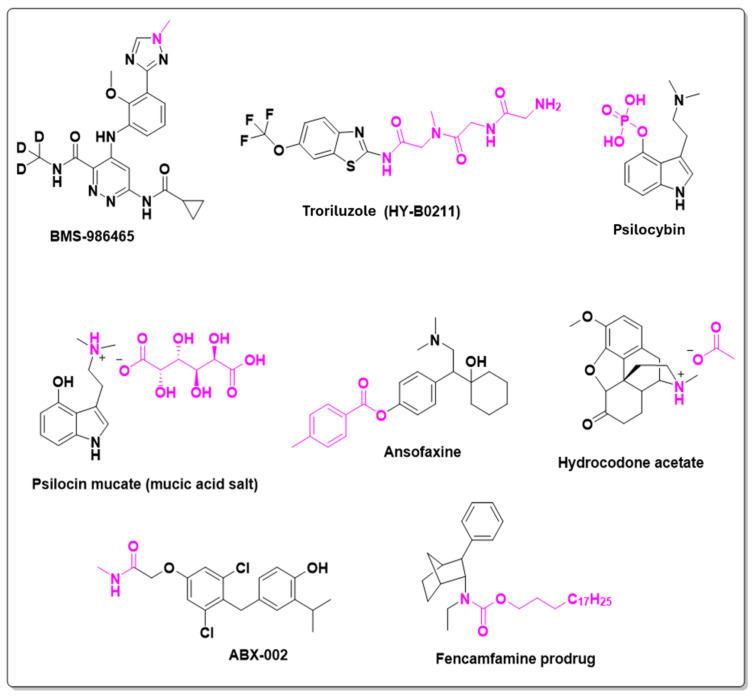
BMS-986465, troriluzole, psilocybin, psilocin mucate, ansofaxine, hydrocodone acetate, ABX-002, and fencamfamine prodrug chemical structures (magenta: moiety group).

Figure 15 shows the chemical structures of BMS-986465, troriluzole, psilocybin, psilocin mucate, ansofaxine, hydrocodone acetate, ABX-002, and fencamfamine as central nervous system prodrugs.

### 2.5. Other Prodrugs

#### 2.5.1. Anti-Inflammatory

Anti-inflammatory drugs are among the most widely used therapeutic classes worldwide. Nonsteroidal anti-inflammatory drugs (NSAIDs) have been integral to human medicine since ancient Greece [79], where infusions made from the bark of Salix trees, a source of salicin, were used as analgesics. More than 5000 years later, in 1897, Felix Hoffmann synthesized Aspirin^®^, an acetylated derivative of salicylic acid. The mechanism of action of Aspirin and other NSAIDs involves the inhibition of cyclooxygenase-1 (COX-1) and cyclooxygenase-2 (COX-2), which are key enzymes in the production of prostaglandins responsible for pain and fever. However, certain pathological conditions, such as cancer, rheumatoid arthritis, and allergies, are characterized by chronic inflammation [80].

Receptor-interacting proteins (RIPs) are a class of proteins with serine/threonine kinase activity. Upon activation, RIPK2 undergoes autophosphorylation and forms lysine-63 (K63)-linked polyubiquitin chains, which promote the activation of the MAPK (mitogen-activated protein kinase) and NF-κB signaling pathways [81]. Dysregulation of the NF-κB pathway is associated with various conditions, including inflammatory diseases, severe pulmonary sarcoidosis, and multiple sclerosis [82]. Recently, this pathway has gained prominence as a target in anti-inflammatory therapies. For instance, GSK2983559 is an orally active and potent inhibitor of receptor-interacting protein 2 (RIP2) kinase. This phosphate prodrug entered Phase I clinical trials in 2019, but the study was terminated due to non-clinical toxicology findings and a reduced safety margin. In 2020, a Phase II clinical study evaluated the efficacy and safety of a new prodrug for managing pain associated with osteoarthritis. Another example, VX150, an pyridine amide derivative, is a highly selective oral inhibitor of NaV1.8 (a sodium channel), which has shown promise in alleviating acute pain [83,84,85].

**Figure 16:**
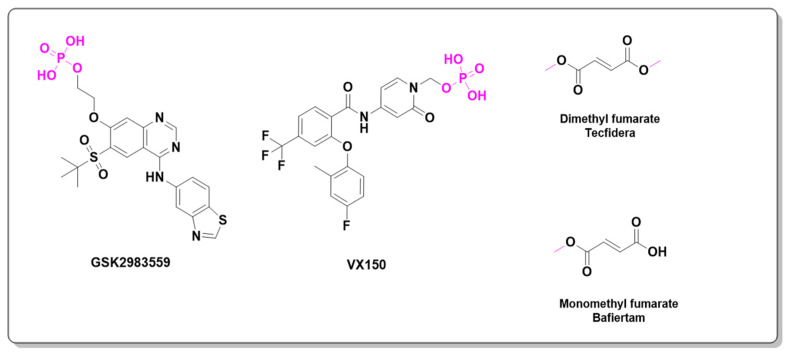
GSK298355, VX150, dimethyl fumarate, and monomethyl fumarate prodrug chemical structures (magenta: moiety group).

Dimethyl fumarate (Tecfidera^®^) and its derivative monomethyl fumarate (Bafiertam™) are prodrugs derived from fumaric acid [86]. A Phase I clinical study evaluated the gastrointestinal tolerability of bioequivalent doses of these drugs and compared their safety and tolerability in healthy subjects following bioequivalent oral regimens. These drugs are indicated for the treatment of multiple sclerosis (MS), and preclinical studies suggest that their mechanism of action involves neuroprotection and immune regulation, which may help mitigate ongoing MS symptoms [87]. Figure 16 shows the GSK298355, VX150, dimethyl fumarate, and monomethyl fumarate prodrug chemical structures.

#### 2.5.2. Immunomodulator

The immune system is a large and complex network that is not yet fully understood. It primarily consists of two components: the innate immune system, which provides an immediate, nonspecific defense, and the adaptive immune system, which offers a more specialized response. These systems comprise various white blood cells, including lymphocytes, neutrophils, mast cells, dendritic cells, natural killer (NK) cells, and T cells, among others. Together, they protect the body against pathogens like bacteria, viruses, and parasites through intricate cellular and molecular pathways.

However, due to genetic predispositions, viral infections, or other triggers, the immune system can sometimes develop autoimmunity, mistakenly attacking the body’s own tissues. This results in the release of significant amounts of pro-inflammatory mediators, leading to autoimmune diseases such as systemic lupus erythematosus [88], Sjogren’s syndrome [89], ankylosing spondylitis [90], polymyalgia rheumatica [91], alopecia areata [92], Crohn’s disease [93], vitiligo, and psoriasis [94].

To manage these autoimmune conditions, various drugs and biopharmaceuticals are available that modulate the immune response and reduce harmful inflammation. Examples include steroidal anti-inflammatories and recombinant drugs such as Tremfya^®^ (guselkumab) and Humira^®^ (adalimumab), as well as cytokine analogs like AVONEX^®^ (interferon beta-1a). Additionally, immunosuppressants such as mycophenolate mofetil and danazol are commonly used to dampen immune reactivity in autoimmune diseases.

A recent development in immunomodulatory therapy is ANX1502, a first-in-class orally available small molecule prodrug inhibitor targeting the classical complement pathway. Developed by Annexon, Inc., ANX1502 entered Phase I clinical trials in 2022. While detailed molecular data for ANX1502 is not yet available, it is currently being evaluated for treating cold agglutinin disease (CAD). CAD is a rare type of autoimmune hemolytic anemia characterized by elevated levels of circulating antibodies that cause the immune system to attack and destroy red blood cells, leading to significant anemia and related complications [95]. This new therapeutic approach represents a promising advance in targeting specific components of the immune system for the treatment of autoimmune diseases. If successful, ANX1502 could characterize a novel option for managing CAD and potentially other complement-mediated autoimmune disorders.

#### 2.5.3. Hormones

Hormones are a class of biomolecules characterized by a core structure known as steroid tetracycle, specifically cyclopentanoperhydrophenanthrene. This structure gives rise to various biomolecules such as testosterone, estrogen, cortisol, and plant-derived compounds like saponins [96,97]. Due to their biological actions, drugs and prodrugs have been developed to either inhibit or agonize steroid receptors. Notable examples include synthetic anabolic steroids like testosterone propionate, female contraceptives such as ethinylestradiol and levonorgestrel, and steroidal anti-inflammatory drugs (corticosteroids) such as dexamethasone, betamethasone, and cortisone. The clinical use of hormones and hormone analogs is fundamental for treating various endocrine disorders, managing hormone deficiencies, and supporting reproductive and metabolic health needs. Hormone therapies, such as testosterone and estradiol, are commonly prescribed for conditions like hypogonadism, menopause, and gender-affirming treatments. Efficacy and patient adherence to these treatments can be significantly enhanced by using prodrug formulations.

One effective prodrug strategy is to employ long-chain carboxylic acid esters, including testosterone enanthate, testosterone propionate, testosterone isocaproate, testosterone decanoate, estradiol valerate, and estradiol cypionate. These esterified hormone forms allow for prolonged and controlled release, typically administered as depot injections. Once injected, these compounds slowly release the active hormone over time as the ester group is gradually cleaved in the body. This method minimizes the need for frequent dosing, providing a more consistent therapeutic effect and improving patient compliance. This depot strategy using fatty acid esters extends beyond hormone therapy and is applied to other drug classes that benefit from controlled release. For instance, antipsychotics like haloperidol palmitate, anti-inflammatory agents such as dexamethasone palmitate, and antibiotics like chloramphenicol palmitate utilize similar ester prodrug approaches. These formulations enhance bioavailability, prolong the duration of action, and reduce dosing frequency, making them valuable tools across multiple therapeutic areas.

In 2019, TesoRx Pharma conducted a Phase I/II clinical study to evaluate the pharmacokinetics of testosterone undecanoate in hypogonadal males. This condition involves insufficient production of testosterone, crucial for male growth and development during puberty, and may also affect sperm production. Testosterone undecanoate is a prodrug containing a long fatty acid chain (undecylic acid), designed for sustained release. However, the study was discontinued due to lack of efficacy.

**Figure 17:**
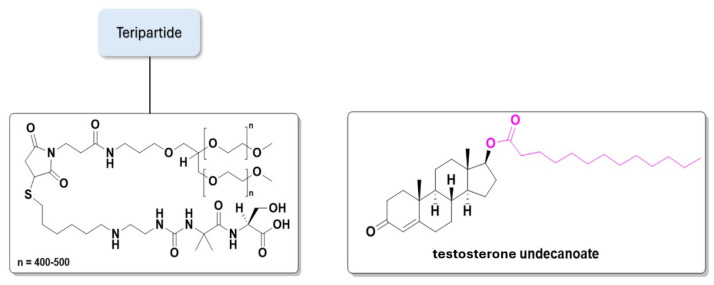
Transcon PTH and testosterone undecanoate prodrug chemical structures (magenta: moiety group).

Similarly, in 2024, TransCon PTH—palopegteriparatide, a prodrug of parathyroid hormone (PTH 1-34)—was entered into a Phase III multicenter clinical trial to investigate its safety, tolerability, and efficacy in adults with hypoparathyroidism, an endocrine disorder. Teriparatide, a recombinant human parathyroid hormone analogue (PTH 1-34), serves as the basis for this drug. Palopegteriparatide binds a methoxy polyethylene glycol via a cleavable link, creating an analogue corresponding to amino acids 1–34 of human PTH. Outcomes from the TransCon study demonstrated a significantly higher proportion of patients achieving normal serum calcium levels and independence from conventional therapy compared to placebo with TransCon PTH. In PaTH Forward, long-term treatment with TransCon PTH maintained a durable response, with 93% of patients achieving independence from active vitamin D and therapeutic calcium levels through week 110 [98,99]. Figure 17 shows the Transcon PTH and testosterone undecanoate prodrug chemical structures.

#### 2.5.4. Sickle Cell Disease

Sickle Cell Disease (SCD) is a genetic blood disorder characterized by abnormal hemoglobin, a crucial protein for oxygen transport. This abnormality causes red blood cells to become rigid and adopt a crescent or sickle shape, leading to numerous complications, including severe pain, anemia, organ damage, and heightened susceptibility to bacterial infections [100,101]. SCD predominantly affects individuals of African, Mediterranean, Middle Eastern, and Indian descent due to genetic predisposition. In the United States, approximately 100,000 people, primarily of African heritage, are affected [102].

Treatment focuses on symptom management and complication prevention through pain relief, blood transfusions, and antibiotics. Hydroxyurea, which boosts fetal hemoglobin production, has proven effective in symptom reduction [103]. In severe cases, bone marrow or stem cell transplants, as well as gene therapy, offer potential cures [104]. Among strategies for discovering new drugs for SCD, augmenting fetal hemoglobin (HbF) is a validated approach [105].

In 1978, sodium butyrate was found to increase histone acetylation levels, leading to elevated fetal hemoglobin production. However, its irregular pharmacokinetics required continuous high-dose infusion (2000 mg/kg/day) and inconvenient intravenous administration, raising concerns about its clinical application [106,107]. Other short-chain fatty acids were also investigated, among which 2,2-dimethylbutyrate showed promising effects both in vitro and in vivo. Clinical trials revealed a modest increase in HbF (0.2 g/dL) [108], but the compound exhibited irregular pharmacokinetics [109,110].

The mode of action of butyrates involves weak inhibition of histone deacetylase (HDAC), affecting γ-globin gene transcription, which induces fetal hemoglobin production. However, butyrates undergo phase II metabolism due to the carboxylic acid group, leading to rapid elimination and reduced therapeutic efficacy. Ionization of the carboxylic acid also hinders absorption, limiting their potential. A common strategy to overcome this involves masking ionizable species through prodrug design. Hydroxamate derivatives were synthesized to optimize pharmacokinetics and pharmacodynamics. Butyrylhydroxamate inhibited HDAC from purified rat liver with an IC50 of 47 µM and increased γ-globin gene expression by up to 5.5-fold in vitro. In transgenic sickle cell mice, a dose of 500 mg/kg/day increased HbF levels [111,112]

Interestingly, vorinostat (SAHA), an HDAC inhibitor approved by the FDA in 2006 for cutaneous T-cell lymphoma, also induced γ-globin gene expression. Vorinostat inhibited HDAC with an IC50 of 0.26 µM and increased γ-globin expression 2.5-fold in vitro at 2.5 µM. Clinical trials with doses of 100–400 mg/day reported increases in HbF ranging from 4.6% to 20.5% [113]. Another prodrug, romidepsin, is a cyclic depsipeptide with non-selective HDAC inhibition. It is bioactivated in vivo, releasing a thiol group that inhibits HDAC. Romidepsin increased γ-globin expression by 7.81-fold at 1 nM in GM979 cells and enhanced F-erythroblast levels in BUF-E colonies from 13.3% to 34.9% at 100 pM. However, no clinical trials for SCD with romidepsin have been reported [112].

Selective HDAC inhibitors are also promising. For example, MGCD-0103, an HDAC-1/-2 inhibitor, induced γ-globin expression, highlighting selective HDAC inhibition as a viable strategy for SCD drug development. Preclinical studies of ACY-957, another selective HDAC inhibitor, showed promising effects [114,115,116]. Enzymes such as HDAC, lysine-specific histone demethylase 1 (LSD1), and DNA methyltransferase (DNMT1) play crucial roles in γ-globin gene regulation. DNMT inhibitors like 5-azacytidine and decitabine induce HbF production but cause severe adverse effects, including myelosuppression, limiting their use [117,118,119]. Approaches using prodrugs, such as S110, have improved stability and efficacy. S110 doubled γ-globin mRNA ratios and increased HbF production by up to 60%, outperforming decitabine in preclinical studies [120].

**Figure 18:**
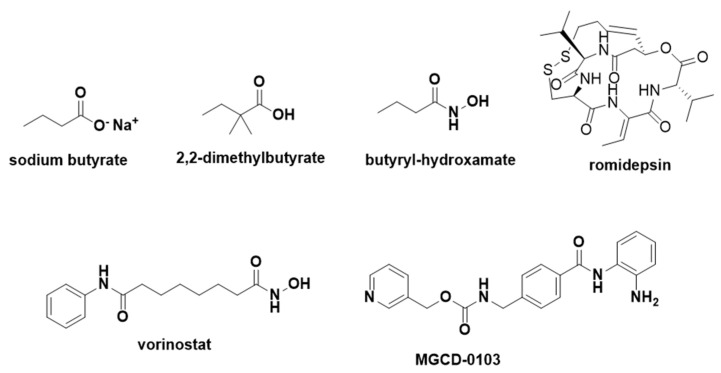
Sodium butyrate, 2,2-dimethylbutyrate, butyryl-hydroxamate, romidepsin, vorinostat, and MGCD-0103 chemical structures.

**Figure 19:**
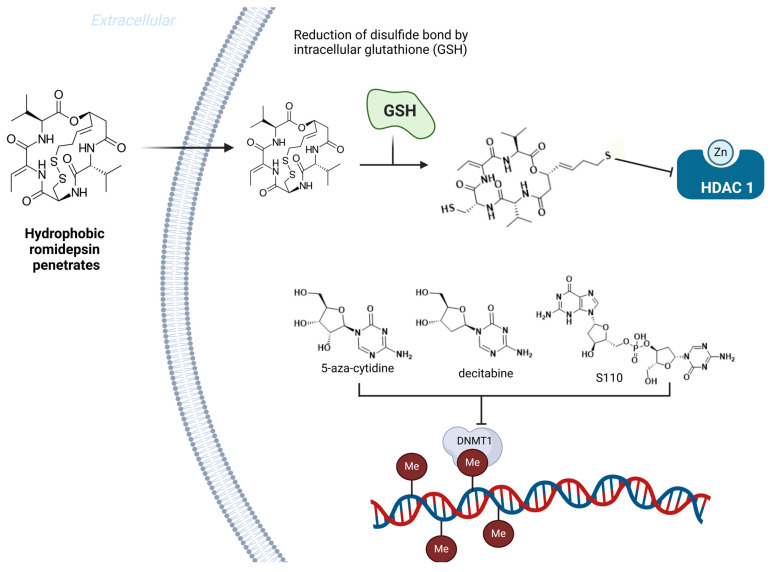
Mechanism of romidepsin activation mediated by GSH and the inhibition of HDAC1 by thiolate formation. DNMT1 inhibitors 5-aza-cytidine, decitabine, and S110.

Ongoing research includes GSK4172239D, a novel DNMT1 inhibitor currently in Phase I clinical trials to evaluate safety, tolerability, and pharmacokinetics in SCD patients, although molecular data remain unavailable [121]. Figure 18 shows the sodium butyrate, 2,2-dimethylbutyrate, butyryl-hydroxamate, romidepsin, vorinostat, and MGCD-0103 chemical structures, and Figure 19 shows the mechanism of romidepsin activation mediated by GSH and the inhibition of HDAC1 by thiolate formation.

#### 2.5.5. Antifungal

Fungi are living organisms that can be either harmless or pathogenic to humans. They exist as molds, yeasts, or a combination of both forms. Fungal infections are classified as either primary or opportunistic and can be local or systemic. Local infections typically affect areas such as the skin, mouth, or vagina, and can occur in both healthy and immunocompromised individuals. In contrast, systemic infections not only impact the skin but also spread to internal organs and are primarily seen in immuno-compromised patients. Antifungal drugs, or antimycotics, treat these infections by targeting unique fungal structures that differ from those of humans or bacteria. These drugs are structurally diverse, including subgroups such as allylamines, azoles, fluoropyrimidines, and polyene macrolides. Allylamines, like naftifine, inhibit squalene epoxidase, essential for ergosterol biosynthesis, a key component of the fungal cell membrane. Azoles, such as voriconazole and posaconazole, inhibit lanosterol demethylase, disrupting the same pathway. Fluoropyrimidines interfere with nucleoside synthesis, while amphotericin B, a polyene macrolide, targets fungal cell membranes [122].

A novel antifungal agent, Fosmanogepix, is a prodrug of Manogepix with broad-spectrum activity against yeasts like *Cryptococcus* and *Candida*, as well as molds, including azole-resistant *Aspergillus*. It targets the Gwt1 enzyme, essential for the localization of glycosylphosphatidylinositol (GPI)-anchored mannoproteins, thereby compromising fungal cell wall integrity. Effective against drug-resistant strains, it has shown activity against *Candida*, *Fusarium*, and *Scedosporium* in animal models, as well as *Aspergillus fumigatus* and *Rhizopus arrhizus* in pulmonary infections. Fosmanogepix has favorable pharmacokinetics, with over 90% oral bioavailability and good tissue distribution, making it a promising treatment for invasive fungal infections. Unlike many antifungals, it has minimal drug interactions and improved tolerability, offering a new option for combating resistant fungi [123].

**Figure 20:**
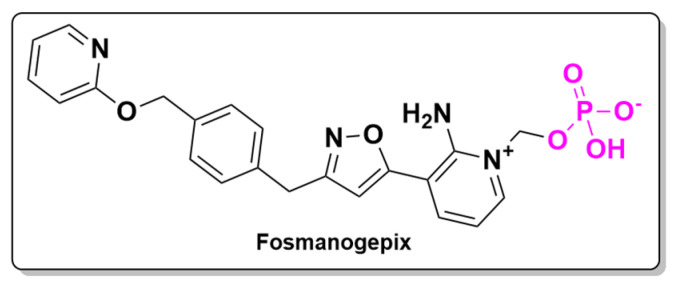
Fosmanogepix prodrug chemical structure (magenta: moiety group).

The active ingredient of this prodrug, Manogepix, was compared to four other antifungal drugs—caspofungin, fluconazole, posaconazole, and voriconazole—in a comparative in vitro study on 90 clinical isolates of *Candida*. Manogepix demonstrated superior activity and potency compared to all the other drugs [124]. The mode of action of this novel drug involves inhibiting inositol acyltransferase Gwt1, an enzyme in the GPI anchor biosynthesis pathway. This inhibition prevents GPI-anchored proteins from maturing within the fungus. Fosmanogepix (Figure 20) has an additional phosphate group at the nitrogen in the heterocycle compared to its active ingredient, Manogepix. Like antibacterial drugs, antifungals urgently require innovation as there is a reported increase in resistance to currently available treatments.

#### 2.5.6. Diabetic Macular Edema

Diabetic Macular Edema (DME) is characterized by the accumulation of excess intraretinal fluid, leading to retinal swelling [125]. This condition arises from two primary changes in retinal circulation: increased vessel permeability and vessel closure. In DME, elevated pressure within retinal vessels causes the endothelial walls to become more porous, leading to fluid leakage into retinal tissue and resulting in edema. At the same time, the closure of vessels impairs the delivery of essential nutrients and oxygen, further exacerbating retinal damage. DME is a leading cause of vision loss and blindness in diabetic patients.

Currently, six primary treatment options are available for managing DME: anti-vascular endothelial growth factor (Anti-VEGF) therapies, topical corticosteroids, laser photocoagulation, pars plana vitrectomy, systemic agents, and surgical interventions. Among these, Anti-VEGF therapies are considered the first-line treatment for DME, aiming to reduce abnormal blood vessel growth and fluid leakage that contribute to retinal swelling [126].

**Figure 21:**
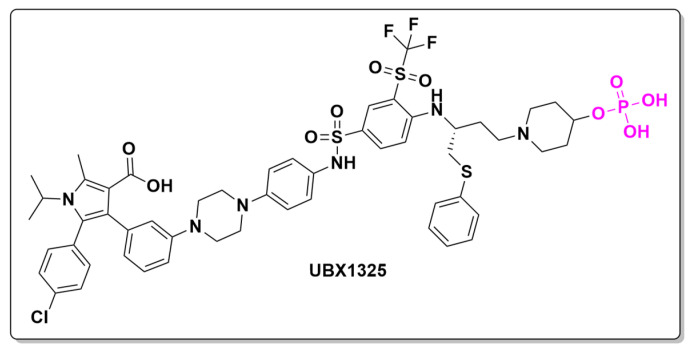
UBX1325 prodrug chemical structure (magenta: moiety group).

As part of our analysis of ongoing clinical trials investigating novel prodrugs for DME, one relevant study was identified. A Phase II trial is currently evaluating the safety, tolerability, and efficacy of UBX1325, a phosphate prodrug administered via intravitreal injection. UBX1325 functions by inhibiting B-cell lymphoma extra-large (Bcl-xL), a protein that promotes cell survival. By targeting Bcl-xL, UBX1325 enhances apoptosis (programmed cell death) in damaged cells, reducing pathological tissue overgrowth and fluid accumulation in the retina. In addition to DME, UBX1325 is also being investigated for other retinal diseases, such as age-related macular degeneration (AMD) and diabetic retinopathy (DR). According to preliminary results from the manufacturer, UBX1325 (Figure 21) has demonstrated promising efficacy and safety profiles, positioning it as a potential alternative or adjunct to Anti-VEGF therapy for DME in the future [127].

#### 2.5.7. Hypertension

Hypertension is one of the leading risk factors for cardiovascular disease, presenting a significant global health and economic burden. Affecting approximately 1.28 billion people worldwide, nearly 46% of cases remain undiagnosed, according to the World Health Organization (WHO) [128]. If untreated, hypertension can lead to severe complications, including cardiovascular disease, stroke, and kidney failure. Many patients with high- or very high-risk hypertension exhibit multiple cardiovascular risk factors, often accompanied by subclinical organ damage or established cardiovascular or renal disease.

The primary classes of antihypertensive medications include beta-blockers, calcium channel blockers (CCBs), angiotensin-converting enzyme (ACE) inhibitors, diuretics, and angiotensin II receptor blockers (ARBs). These classes have long been the pillar of hypertension management. To enhance the bioavailability and extend the duration of ACE inhibitors, several ester prodrugs—such as ramiprilat, quinalaprilat, and imidaprilat—were developed, offering higher oral bioavailability than their active forms [129]

In recent years, advancements in cardiovascular drug development have introduced new therapeutic targets for hypertension and related conditions. For example, KAR5585 (rodatristat ethyl) has emerged as a promising first-in-class oral tryptophan hydroxylase 1 (TPH1) inhibitor. It is currently under investigation for the treatment of pulmonary arterial hypertension (PAH), a condition where elevated serotonin levels contribute to vascular remodeling and increased pulmonary pressure [130,131]. In a Phase 1 clinical trial, KAR5585 was assessed for its pharmacokinetics through single and multiple ascending doses in healthy subjects. By targeting serotonin biosynthesis, KAR5585 aims to lower serotonin levels in peripheral tissues, potentially alleviating PAH symptoms.

**Figure 22:**
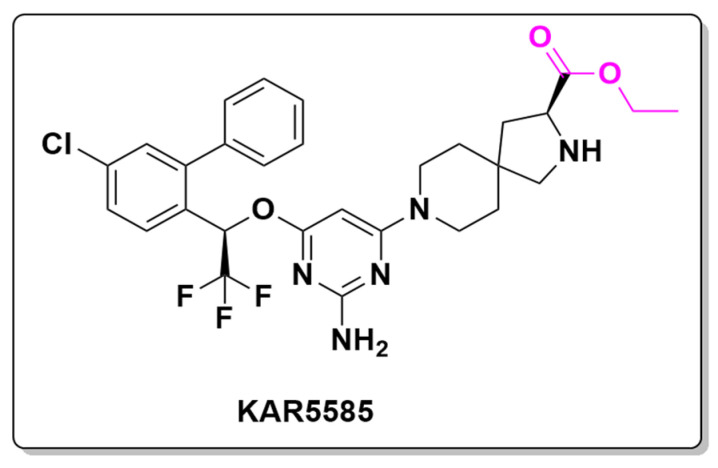
KAR5585 prodrug chemical structure (magenta: moiety group).

Structurally, KAR5585 incorporates an ester moiety that enhances cell permeability by preventing premature deprotonation, facilitating cell membrane penetration. Once inside the cell, the ester group undergoes hydrolysis, releasing the active form of the drug. Additionally, KAR5585 (Figure 22) includes a 2,8-diazaspiro [4.5]decane moiety, which has been reported as a scaffold for epoxide hydrolase inhibition, a novel approach for hypertension treatment [132].

In parallel with KAR5585, other clinical trials have explored alternative treatments for hypertension. These studies reflect the evolving landscape of cardiovascular drug research, where novel mechanisms—such as serotonin modulation inhibition—are being explored to provide more targeted treatments for complex conditions like hypertension.

#### 2.5.8. Toxicity Reduction

Mitochondrial diseases, particularly those involving mitochondrial complex I dysfunction, are severe and often life-threatening disorders that primarily impact cellular energy production. The mitochondrial respiratory chain comprises four complexes (I–IV) essential to oxidative phosphorylation, the process by which ATP—the cell’s primary energy currency—is generated. Complex I, also known as NADH oxidoreductase, is the largest and one of the most critical complexes in this chain [133]. Dysfunction in complex I is a major cause of mitochondrial diseases, including Leigh syndrome, a neurodegenerative disorder that typically manifests in infancy or early childhood and leads to progressive neurological decline [134].

Leigh syndrome and other mitochondrial complex I deficiencies result in significantly reduced ATP production, impairing tissues with high energy demands, such as the brain, heart, and muscles. Currently, there are no effective treatments available for these disorders, and management often focuses on providing symptomatic relief. An emerging therapeutic approach involves using succinate, a crucial metabolite in the tricarboxylic acid (TCA) cycle and a substrate for mitochondrial complex II (succinate dehydrogenase). Theoretically, bypassing the dysfunctional complex I by delivering succinate directly to complex II could help restore cellular energy production. However, succinate has limited therapeutic potential because it does not easily cross cell membranes due to its low permeability.

To overcome this barrier, researchers are developing succinate prodrugs that can permeate cell membranes and release succinate within the cell. One promising candidate is NV354, a succinate prodrug developed by Abliva, Inc. NV354 includes a sulfur-containing ester moiety that enhances cell membrane permeability. Once inside the cell, this ester group is cleaved, releasing active succinate, which can then be utilized by complex II to stimulate ATP production, effectively bypassing the dysfunctional complex I [135]. A preclinical study by Piel et al. [136] tested NV354 in a rat model of fluoroacetate-induced toxicity, a condition that impairs mitochondrial function. While the treatment produced a modest effect in heart tissue, it yielded more promising results in the brain, suggesting that NV354 effectively crosses the blood–brain barrier and can restore mitochondrial function in neurological tissues. This is particularly significant, as mitochondrial diseases often result in severe neurological impairments.

NV354 has demonstrated good oral bioavailability, stability in plasma, and an ability to cross the blood–brain barrier—critical attributes for a drug targeting mitochondrial disorders. In 2018, NV354 entered Phase II clinical trials to evaluate its potential for treating complex I-related mitochondrial diseases in humans.

**Figure 23:**
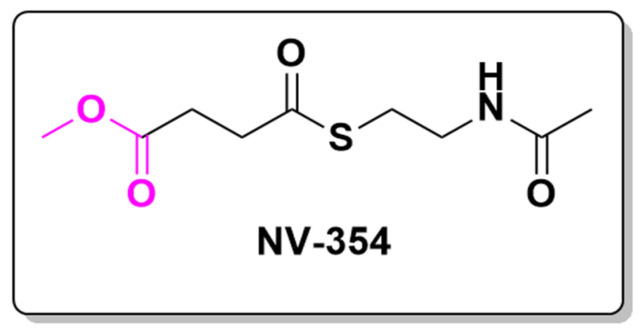
Chemical structure of NV-354 prodrug (magenta: moiety group).

The prodrug’s structure includes an ester promoiety that facilitates the crossing of the blood–brain barrier by preventing the formation of a deprotonated ionic form, which would otherwise hinder its passage. If successful, NV354 could represent a breakthrough in the treatment of these currently untreatable mitochondrial diseases by directly addressing the bioenergetic deficit caused by complex I dysfunction. This strategy, focused on metabolic correction via prodrugs like NV354 (Figure 23), offers new hope for future mitochondrial disease therapies and could significantly improve outcomes for patients with complex I deficiencies.

## 3. Conclusions and Future Directions

In summary, the design and development of prodrugs, as an old strategy, continues to be a powerful tool for advancing treatments across a wide range of clinical conditions, including cancer, bacterial infections, hypertension, and more, to reach therapeutic efficacy, with more selectivity and less toxicity by optimizing pharmacokinetic parameters, such as lipophilicity (logP), water solubility, and oral bioavailability, and delivering the drug to a specific target, specially as personalized medicine.

To address these challenges, medicinal chemistry can explore non-traditional promoieties, including boron-containing groups, peptides, and cleavable linkers that are activated by enzymatic or environmental triggers, to refine and expand prodrug applications. As such, the prodrug approach will remain an important strategy in future drug discovery, contributing to the design of safer and more effective treatments for complex diseases and individual patient needs.

## Figures and Tables

**Table 1:** Antineoplastic prodrugs and their clinical trial phase status.

Prodrug (Moiety Group Is in Magenta)	Name	Condition	Phase	Promoiety
** 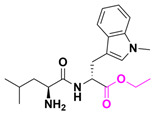 **	Indoximod (NLG802)	Solid tumor	I	Ester
** 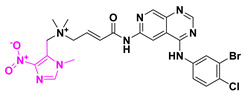 **	Tarloxotinib hydrobromide	Solid tumors, head and neck cancers.	II	Bromide salt
** 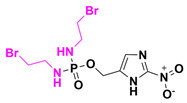 **	Evofosfamide	Solid tumors	I	Phosphamide
** 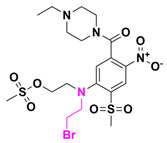 **	CP506	Solid tumor	I/II	Hypoxia-activated prodrug
** 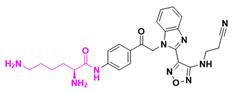 **	Lisavandibulin (BAL101553)	Solid tumor/ Glioblastoma	I/II	Amide
** 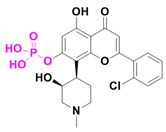 **	Alvocidib (TP1287)	CDK9 inhibitor/Advanced solid tumor	I	Phosphate

**Table 2:** Clinical ADC studies from January to October 2024.

NCT ID	ADC ID	Target	Status	Phase	Sponsor
NCT06561607	TQB2102	Breast cancer with low HER2 expression in the recurrent Metastatic stage	Not yet recruiting	1	Chia Tai Tianqing Pharmaceutical Group Co., Ltd.
NCT06496490	TQB2102	Locally advanced or metastatic non-small cell lung cancer with HER2 gene abnormality	Recruiting	2	Chia Tai Tianqing Pharmaceutical Group Nanjing Shunxin Pharmaceutical Co., Ltd.
NCT06431490	TQB2102	HER2-positive Biliary Tract Cancer	Recruiting	1/2	Chia Tai Tianqing Pharmaceutical Group Nanjing Shunxin Pharmaceutical Co., Ltd.
NCT06555744	ZW191	Folate Receptor Alpha for Advanced Solid Tumors	Recruiting	1	Zymeworks BC Inc.
NCT06555263	Luveltamab Tazevibulin— STRO-002	Advanced or metastatic non-small cell lung cancer expressing FOLR1 (FolRα)	Recruiting	2	Sutro Biopharma, Inc.
NCT06549816	Sigvotatug vedotin SGN-B6A	Advanced solid tumors	Recruiting	1	Seagen Inc.
NCT06545617	BAT8006	Folate receptor α (FRα). Subjects with solid tumors.	Not yet recruiting	1	Bio-Thera Solutions
NCT06533826	Datopotamab deruxtecan and Trastuzumab deruxtecan	HER2-low locally advanced unresectable or metastatic breast cancer (MBC)	Not yet recruiting	2	Ana C Garrido-Castro, MD
NCT06519370	FDA018-ADC	Locally recurrent inoperable or metastatic Triple-negative Breast cancer (TNBC) who are resistant to or recurring during or after taxane therapy.	Recruiting	3	Shanghai Fudan-Zhangjiang Bio-Pharmaceutical Co., Ltd.
NCT06509997	MRG003 combined with Dalpicicilip posterior line	Recurrent/metastatic CDKN2A gene variant head and neck squamous cell carcinoma (HNSCC)	Not yet recruiting	2	Lei Liu, West China Hospital
NCT06465069	LY4052031 (enfortumab vedotin-ejfv)	Anti-nectin-4 advanced or metastatic solid tumors including urothelial cancer.	Recruiting	1	Eli Lilly and Company
NCT06457997	PHN-010	Advanced solid tumors.	Recruiting	1b	Pheon Therapeutics
NCT06453044	Polatuzumab vedotin	Relapsed or Refractory grade 1-3a Follicular Lymphoma	Recruiting	2	City of Hope Medical Center
NCT06440005	AGX101	Advanced Solid Tumors	Recruiting	1	Angiex, Inc.
NCT06384807	PBI-410	Advanced Solid Tumors	Recruiting	1/2	Biohaven Therapeutics Ltd.
NCT06362252	Ifinatamab deruxtecan (I-DXd), the ADC in combination with immune checkpoint inhibitor (ICI) atezolizumab with or without carboplatin	Extensive stage-small cell lung cancer (ES-SCLC) in the first line (1L) setting.	Recruiting	1/2	Daiichi Sankyo
NCT06341400	RC48 Combined with Toripalimab	Platinum-intolerant bladder cancer patients. tumor cells	Recruiting	1/2	Zhujiang Hospital
NCT06265727	CRB-701	Solid tumors that express nectin-4.	Recruiting	1/2	Corbus Pharmaceuticals Inc.
NCT06238479	LY4101174	Humanized immunoglobulin G1 (IgG1) Fcg-silent monoclonal antibody directed against the cell surface adhesion molecule and tumor-associated antigen (TAA) nectin-4 (PVRL4) conjugated, via maleimide-beta-glucuronide poly-sarcosine linkers, to the camptothecin analog and topoisomerase 1 inhibitor exatecan, with potential antineoplastic activity.	Recruiting	1	Eli Lilly and Company
NCT06234423	CUSP06-1001	Platinum-Refractory/Resistant Ovarian Cancer and Other Advanced Solid Tumors	Recruiting	1	OnCusp Therapeutics, Inc.
NCT06210490	Disitamab Vedotin	Adjuvant Treatment of HER2 Overexpressing UTUC Patients with high risk factors for recurrence after radical surgery	Not yet recruiting	2	Peking University First Hospital
NCT06210815	HLX42	EGFR-targeting ADC, for cetuximab or TKI resistant cancer	Recruiting		Shanghai Henlius Biotech
NCT06144723	HS-20105	Targeting Trop-2 advanced solid tumors	Not yet recruiting	1/2	Hansoh BioMedical R&D Company
NCT06132958	Sacituzumab Tirumotecan (MK-2870)	Endometrial cancer (EC) that have previously received treatment with platinum-based therapy.	Recruiting	3	Merck Sharp & Dohme LLC
NCT06112704	HS-20093	Advanced esophageal carcinoma and other solid tumor	Recruiting	2	Hansoh BioMedical R&D Company

## Data Availability

All data are available in the manuscript.

## Supplementary Materials

The following supporting information can be downloaded at: www.mdpi.com/article/10.3390/ph18020210/s1, Table S1: Clinical trials data.
