# Supplementary material for: Current Trends in Clinical Trials of Prodrugs
Source: Pharmaceuticals (Basel). 2025 Feb 4;18(2):210. doi: 10.3390/ph18020210 (PMC11859331; doi:10.3390/ph18020210)
Supplement: Supplementary file 1 [file pharmaceuticals-18-00210-s001.zip › pharmaceuticals-3457137-supplementary.pdf]

Table S1. Clinical Trials Data

NCT ID	Compound Name	Indication	Year
NCT02674581	BMS-663068 (fostemsavir)	Antiviral	2018
NCT06157242	Xerborbactan - Access of PK of QPX7831	Antibiotics	2024
NCT05572190	Oral Overdose Protected Hydrocodone (Hydrocodone acetate controlled by hydrocodone bitartrate hemipentahydrate)	Pain Management	2023
NCT04315324	AKR1C3 target prodrug (OBI3424)	Refractory Leukemia	2024
NCT04627532	PF-00835231 phosphate	COVID-19	2021
NCT04638803	Fencamfamine prodrug	CNS Disorders	2021
NCT05528315	ABX-002	CNS Disorders	2021
NCT03597438	Succinate prodrugs (reducing toxicity)	Various Diseases	2024
NCT05469490	NLG802 - Indoximod prodrug	Advanced Solid Tumors	2022
NCT02746237	KAR5585	Hypertension	2016
NCT04857996	UBX1325	Diabetic Macular Edema	2024
NCT05521269	ANX1502	Aglutinin Diseases	2023

NCT02612285	SNX5422	Null Cancers (HSP90 Inhibitor)	2018
NCT02454842	Tarloxotinib bromide (TH4000)	Cancer	2023
NCT04261413	RS0139	Lung Cancer	2023
NCT04710407	Terbipenem HBr (SPR994)	Antibiotics	2021
NCT03823989	PROMITIL Intravenous (Mitomycin Liposome)	Anticancer Agent	2022
NCT04022473	Tecfidera (Dimethyl Fumarate)	Multiple Sclerosis (Anti-inflammatory)	2020
NCT03358407	GSK983559	Anti-inflammatory	2020
NCT05614258	ADG206 Antibody Prodrugs	Cancer	2023
NCT02598687	Evofostamide (TH302)	Anticancer	2016
NCT04701203	TransCon PTH	Various Diseases	2024
NCT04969835	AVA6000	Solid Tumor (FAPa - Doxorubicin)	2023
NCT02750761	Tedizolid Phosphate	Antibiotic	2019
NCT05700734	MK-8510	HIV (Antiviral)	2023
NCT06074497	KGX101	IL12 Inhibitor (Cancer)	2023
NCT03530917	Rozutolimod	Viral Infection	2020
NCT05955586	Fobrepodacin SPR720	Antibiotic	2024
NCT02271412	Ansofaxina LY03005	Antidepressant	2015
NCT04019717	AT-527	HCV	2020

NCT05488678	VNRX-714 (Ledaborbactam)	Various Diseases	2023
NCT02660424	VX-150	Anti-inflammatory (Osteoarthritis)	2020
NCT02595203	Afabicin	Antibiotic	2016
NCT04954599	CP-506	Cancer	2014
NCT02895360	Lisavandibulin BAL101553	Cancer	2023
NCT03426605	LAM003	Leukemia	2024
NCT03604783	TP1287 (CDK9 Inhibitor)	Cancer	2024
NCT06035900	Psilocybin Mucato Sal	Neuropsychiatric Disorders	2023
NCT03408080	Troriluzole	Spinocerebellar Ataxia	2024
NCT04248426	ATI-2173 (Fosclevudive Alafenamina)	Antiviral - HBV Inhibitor	2021
NCT05129865	LYT-300 (Allopregnanolone)	Neuroactive Steroid	2023
NCT04939441	Tenofovir Alafenamide	Antiviral	2023
NCT06144697	BMS986465	Neuroinflammation	2024
NCT03335254	Testosterone Undecanoate	Various Diseases	2019
NCT05660265	GSK4172239D	Sickle Cell	2023
NCT05365659	IKS03	Lymphoma	2023
NCT04866641	T1201 (Irinotecan Prodrug)	Cancer	2023
NCT05421858	Fosmanogepix	Candida	2024
